# Developing a Single‐Cell Spatial Transcriptomics Workflow for In Vivo Evaluation of Implanted Biomaterials

**DOI:** 10.1002/advs.202513242

**Published:** 2026-02-15

**Authors:** Alex H.P. Chan, Yunfei Hu, Billie Pardavi, Xueying Xu, Angus J. Grant, Steven G. Wise, Lipin Loo, Richard P. Tan

**Affiliations:** ^1^ School of Medical Sciences Faculty of Health and Medicine Charles Perkins Centre University of Sydney Sydney New South Wales Australia; ^2^ School of Life and Environmental Sciences Faculty of Science Charles Perkins Centre University of Sydney Sydney New South Wales Australia

**Keywords:** bioengineering, biomaterials, in vivo evaluation, polycaprolactone, spatial transcriptomics

## Abstract

In vivo evaluation of biomaterials largely relies on histology to assess biocompatibility and foreign body responses. While effective for capturing end‐stage outcomes, these methods offer limited insight into the cellular mechanisms driving tissue remodeling, hindering efforts to rationally design better biomaterials. Transcriptomics has revolutionized our understanding of gene activity driving cellular function, yet remains underutilized in biomaterial evaluation. Recent advances in high‐resolution spatial transcriptomics now enable precise mapping of gene expression within tissue, offering detailed insight into cellular states and spatial organization. To align biomaterial research with advances in spatial biology, we develop a bioinformatics workflow for the Xenium platform to analyze in vivo responses to implanted materials. Applying this workflow to evaluate electrospun polycaprolactone (PCL) scaffolds implanted subcutaneously in mice, we identify spatially distinct macrophage and fibroblast subpopulations with unique gene expression profiles. Spatial analyses show shared phenotypic features between co‐localized macrophages and fibroblasts, oriented from the scaffold body to its surface. Gene ontology linked these spatial transitions to functional roles, with immune cell recruitment occurring within the scaffold and fibrosis at the surface. These transitions were not detectable by histology, highlighting spatial transcriptomics as a powerful approach for uncovering cellular dynamics and enabling better biologically‐informed design of biomaterials.

## Graphical abstract

This work establishes a standardized, high‐resolution spatial transcriptomics workflow for characterizing host responses to biomaterial implants. Using the 10× Xenium platform, the pipeline integrates spatial mapping, subclustering, and functional enrichment to resolve immune and stromal cell organization at single‐cell resolution, enabling reproducible, systems‐level evaluation of biomaterial performance and biocompatibility.

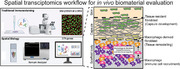

## 1 Introduction

Developing a new biomaterial ultimately requires evaluating its performance in vivo. This is a crucial iterative step in its design process and often serves as the final critical milestone in its translation into medical devices and tissue‐engineered constructs. Following implantation in animals and/or humans, traditional evaluation approaches leverage histological techniques to assess the extent of foreign body responses and local tissue remodeling. Histopathology stains like H&E and Masson's Trichrome are commonly used for broadly assessing tissue remodeling processes like implant fibrosis [1], while immunohistochemistry stains like CD68 and vimentin track specific cell types [2, 3], such as macrophages and fibroblasts, to inform cell‐specific biological responses. These methods remain the dominant metrics for assessing biocompatibility and are widely accepted as both commercial and clinical standards for determining whether a biomaterial integrates or adversely reacts with the body [4]. While these stains highlight key biological outcomes, they provide limited insight into the cellular mechanisms underlying the biological process. As a result, when a biomaterial fails or demonstrates poor biocompatibility, this lack of mechanistic understanding impedes efforts to optimize or redesign the material, as the underlying causes of failure remain unidentified. This has led the field of bioengineering research to rely largely on iterative testing approaches, rather than redesigns biologically‐informed by a detailed understanding of tissue‐ and application‐specific failure mechanisms. A better understanding of the in vivo molecular mechanisms of an implant's failure would transform biomaterial development, providing a targeted optimization workflow to enhance biocompatibility.

The limitations of histological analysis are primarily due to the restricted number of markers that can be visualized simultaneously on a single slide and their spatial resolution constraints at the single‐cell level [5]. Advances in transcriptomics technologies and analysis has revolutionized our understanding of complex and interconnected biological processes by enabling a comprehensive analysis of transcriptional gene activity that drives cell behavior [6, 7]. Profiling large sets of RNA transcripts has uncovered novel cell types and new signaling pathways, providing advances in various fields of medical research through new insights into disease mechanisms, cellular functions, and therapeutic targets [8]. However, despite its transformative impact, transcriptomics has not yet been fully integrated into biomaterial design and evaluation. Genetic analyses are typically performed on separate animal cohorts from those used for histology, making it difficult to correlate cellular structure with gene expression. Common approaches use PCR and, more recently, advanced techniques such as single‐cell sequencing to identify global gene expression patterns and distinct cell populations [9]. However, these methods often employ bulk‐analysis approaches, which mask local variations in biological response. A single biomaterial can exhibit large heterogeneity in architecture, surface chemistry, and tissue contact across its surface, each of which can independently influence local cell behavior [10]. Spatially resolved gene expression that captures changes driven by these local factors would be far more beneficial and relevant for biomaterial evaluation. This would better allow researchers to identify localized effects, optimize material design, and address specific biological interactions not visible in bulk analysis.

Advances in high‐resolution spatial biology techniques have now made it possible to combine spatial imaging with transcriptomic data. Recent studies have combined scRNA‐seq with the amplification‐based 10x Visium spatial transcriptomic to investigate specific biological questions, such as wound healing and fibrosis in biomaterial responses [11, 12]. While these works provide valuable biological insights, they focus on targeted hypotheses, operate at multi‐cell resolution, and do not provide a standardized, high‐resolution workflow that is adaptable for single‐cell spatial analysis across diverse biomaterial contexts. Technologies like the 10x Genomics Xenium platform allow researchers to precisely localize gene expression within cross‐sections of paraffin‐embedded tissue, providing a detailed view of cellular functions and interactions in situ, using sections already compatible with traditional histology analysis, and applying histological analysis to the same tissue [13]. The Xenium platform also offers the advantage of gene expression data at single‐cell resolution, which integrates with extensive online repositories and annotation databases compatible with a wide range of existing bioinformatics analysis tools. To our knowledge, the Xenium platform has not yet been used to map gene expression across foreign body remodeling in vivo to implanted biomaterials. As technology and analysis tools continue to improve, developing a framework that integrates spatial transcriptomics into biomaterial evaluation could uncover new pathways and cellular targets critical to biomaterial design.

In this study, we developed a standardized and reproducible bioinformatics workflow tailored to readouts from the 10× Genomics Xenium platform, enabling high‐resolution single‐cell spatial transcriptomics analysis for in vivo biomaterial evaluation. Using formalin‐fixed paraffin‐embedded tissue of electrospun PCL scaffolds subcutaneously implanted into C57BL6 mice, we demonstrate how spatial transcriptomics can provide new insights into the foreign body response. Xenium high‐dimensional datasets were first assessed to ensure that scaffold implantation did not compromise transcript capture, processing, or visualization. Using Seurat, we implemented an integrated and subclustering analysis approach to segregate gene profiles and cell phenotypes at single‐cell resolution. The readouts from these methods were shown to be compatible with third‐party computational tools, including cell annotation and gene ontology databases, and orthogonally validated against established biological benchmarks using traditional histology. Key features of this workflow included spatial mapping relative to scaffold structures, custom colocalization metrics, trajectory inference to visualize potential directional cell‐state gradients, and subcluster‐level functional annotation. Our workflow revealed distinct macrophage and fibroblast subpopulations with unique localizations within PCL scaffolds. By integrating these computational tools into a stepwise pipeline, the approach is broadly adaptable for biomaterial evaluation, providing a framework that can be potentially applied to new materials, tissues, or experimental designs. Custom computational analysis of spatial colocalization identified a directional gradient of transcriptional states between macrophages and fibroblasts, reflecting shared phenotypic features along the scaffold–capsule interface that are not easily observed with conventional histology. However, while our spatial transcriptomics workflow provides detailed, single‐cell resolution of local tissue responses to biomaterials, it is limited to sampled regions and does not capture systemic or distal effects of implants. Comprehensive evaluation of biomaterial biocompatibility should therefore integrate high‐resolution spatial analyses with traditional histology and transcriptomics across multiple tissues and organs to fully assess both local and systemic responses. These findings showcase a robust and reproducible framework for data acquisition, preprocessing, spatial mapping, and downstream single‐cell gene expression analysis. By complementing and enhancing existing biomaterial evaluation methods, spatial transcriptomics has the potential to provide deeper insights into the foreign body response driving implant failure and to inform the design of next‐generation biomaterials.

## 2 Methods

### 2.1 PCL Scaffold Manufacture

PCL scaffolds were fabricated using the electrospinning technique (IME Medical Electrospinning, Waalre, Netherlands). The PCL solution was made by dissolving PCL in 1,1,1,3,3,3‐hexafluoroisopropanol (HFP) at a concentration of 10% w/v, followed by overnight stirring at room temperature. The prepared solution was then loaded into a syringe (Terumo Corp., Tokyo, Japan) and dispensed at a rate of 4 mL h^−1^ through a 20‐gauge needle. A voltage of 20 kV was applied, with a 16 cm air gap between the needle and a stainless steel drum (10 cm diameter) rotating at 500 rpm. The electrospun scaffolds were collected from the drum, washed, air‐dried to eliminate any remaining solvent, and then cut into 6 mm diameter circular discs using a biopsy punch.

### 2.2 Subcutaneous Implant

The University of Sydney Animal Ethics Committee approved this study under protocol 2024/2438. All experiments adhered to the Australian Code of Practice for the Care and Use of Animals for Scientific Purposes. C57BL/6 male mice (9–10 weeks old, 25 ± 2 g), sourced from Animal BioResources (Moss Vale, NSW, Australia), were used for the study. The in vivo evaluation of PCL scaffolds was performed using a subcutaneous mouse implantation model as previously reported [14]. In summary, each mouse had one scaffold implanted subcutaneously into their back for a period of 14 days, after which the mice were euthanized, and samples were collected for histological and Xenium analysis.

### 2.3 Tissue Processing and Histological Staining

The explanted tissue‐scaffolds were fixed overnight at room temperature in 4% paraformaldehyde. The samples were then dehydrated through a graded ethanol series and xylene, embedded in paraffin, and sectioned at a thickness of 5 µm. For histological staining, the sections were deparaffinized, rehydrated, and stained with Masson's trichrome. For immunohistochemistry, primary antibodies were used against CD68 (1:500, Abcam, ab125212) for macrophages, vimentin (1:200, Abcam, ab45939) for fibroblasts, and CD31 (1:100, Abcam, ab182981) for endothelial cells. The sections were counterstained for nuclei with mounting media containing DAPI (Sigma, F6057) and imaged using a Zeiss AxioScan microscope at 20× magnification.

### 2.4 Spatial Transcriptomics

Spatial transcriptomics experiments were carried out with Xenium analyzer (10× Genomics) with the mouse tissue at lassing panel. Paraffin‐embedded tissues were prepared on manufacturer slides 24 h before sample preparation for the Xenium analyzer. Nuclease‐free water was used to float 5 µm sections for collection on manufacturer slides, then slides were allowed to air dry in 60°C oven for 45 min then overnight in a desiccator. Slide treatment continued according to manufacturer instruction without alteration before analysis on the Xenium instrument.

### 2.5 Downstream Analysis

Reads from Xenium analyzer were compiled and merged using Seurat (v5.2, https://satijalab.org/seurat) [15] installed on R Studios (v4.4.3), related R packages used in analysis include: tidyverse, patchwork, dplyr, ggplot2, and circlize. Standard workflow for dimensional reduction was conducted by starting with principal component analysis, where 15 principal components were identified. These components were used for Uniform Manifold Approximation and Projection (UMAP) dimensional reduction, neighbor distance and clustering (resolution 0.8, Louvain algorithm). To visualize the data, the Seurat function “DimPlot” was used to visualize UMAP data and “FeaturePlot” was used to visualize the expression of a single gene within the UMAP. Further analysis was completed with Monocle 3 to generate trajectories of cell differentiation and pseudotime analysis [16].

### 2.6 Cell Annotation

From the initial UMAP, clusters were identified. “FindMarkers” Seurat function was used to identified highly expressed genes within each cluster, cutoff parameters for these genes were that at least 50% of cells within that cluster had to express that gene and log_2_FC greater than 0.25. The list of genes from each cluster was analyzed using Enrichr and the CellMarker 2024 database [17] to annotate clusters.

### 2.7 Gene Ontology Analysis

Following annotation of clusters, macrophages and fibroblast clusters were further subclustered using the same parameters for UMAP dimensional reduction, neighbor distance and clustering (resolution 0.8, Louvain algorithm). The new subclusters were mapped back on the image for analysis. After image analysis for cell distance from scaffold surface (outlined below), subclusters were classified into either within the scaffold body or the capsule. “FindMarkers” Seurat function was used to identified highly expressed genes within these subclusters, cutoff parameters for these genes were that at least 50% of cells within that cluster had to express that gene and log_2_FC greater than 0.25. Gene ontology analysis was conducted using SRplot [18].

### 2.8 Image Analysis

Images generated from Xenium Explorer 3 (10× Genomics) for a subset of macrophages and fibroblasts were analyzed for distances from the edge of the scaffold and cluster neighbors. For distances of cells, perpendicular distances of each cell to the surface of the scaffold were measured. Negative values indicate inside the scaffold, positive values indicate outside the scaffold. The boundaries of the scaffolds were determined manually with the aid of Masson's Trichrome staining. This process was automated with Python, code is available at https://github.com/ahpchan/STimplant.

For co‐localization, sub‐clusters of cells that are significantly more present in the PCL group relative to skin were included in the analysis. In each image, all cells from one cluster had a 25 µm circle drawn around them. Within each of these circles, counts of other subcluster cells were measured to determine the number of neighboring cells for that subcluster. This was repeated for each subcluster of cells. This process was automated with Python, code is available at https://github.com/ahpchan/STimplant.

### 2.9 Statistical Analysis

Analyses were conducted using GraphPad Prism 10 (GraphPad Software, San Diego, California). Data are presented as mean ± standard error of the mean (SEM). Statistical significance was determined using Student's *t*‐test for comparisons between skin and PCL. A significance level of *p* < 0.05 was considered statistically significant. Symbols ^*^ and ^**^ indicate *P*‐values less than 0.05 and 0.01, respectively.

### 2.10 Ethics Approval Statement

The University of Sydney Animal Ethics Committee Approved This Study Under Protocol 2024/2438. All experiments adhered to the Australian Code of Practice for the Care and Use of Animals for Scientific Purposes.

## 3 Results and Discussion

### 3.1 Quality Metrics of Spatial Transcripts

**FIGURE 1:**
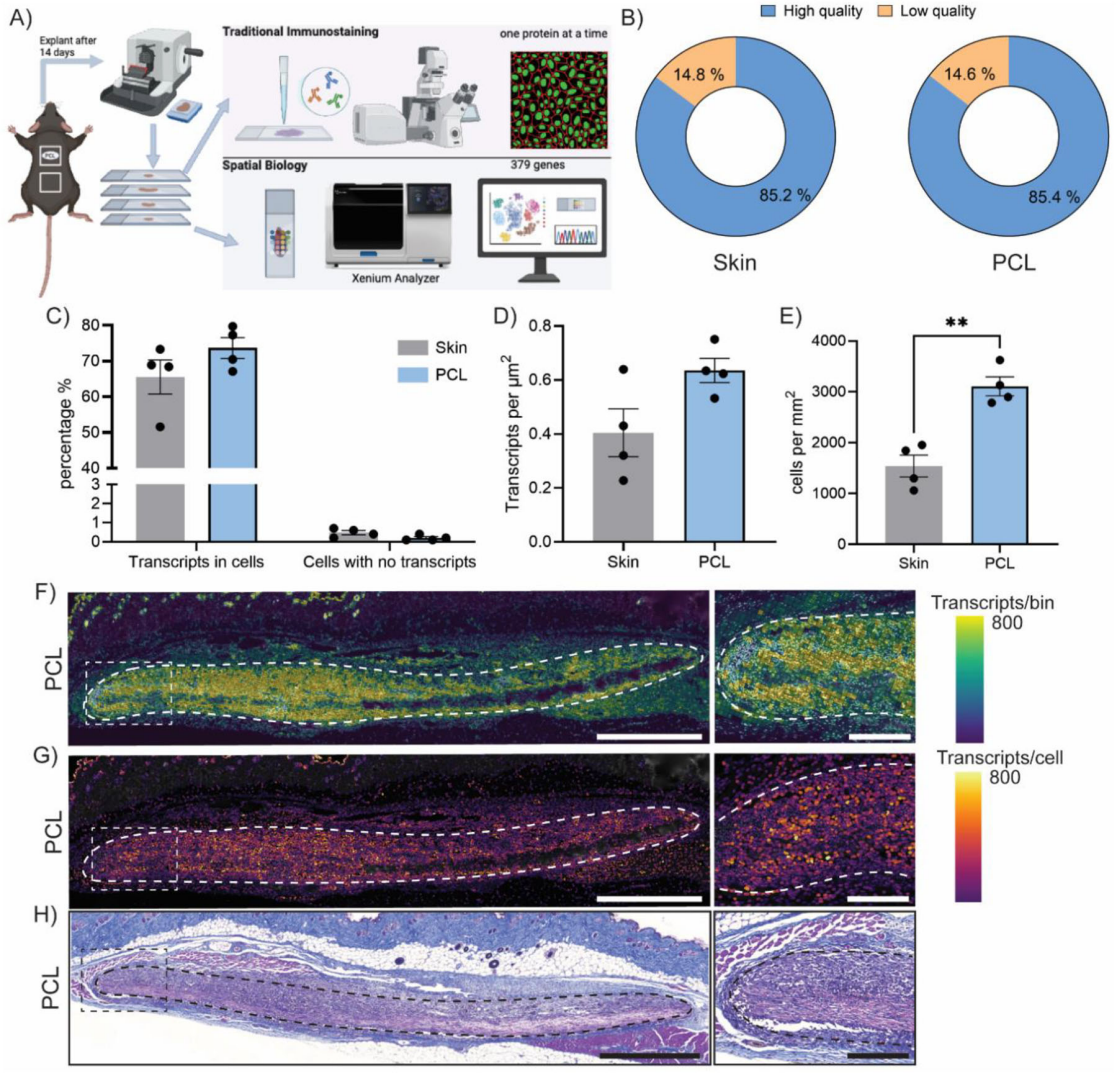
Spatial transcriptomic profiling of skin tissue implanted with electrospun PCL scaffolds. (A) Schematic of experimental design. (B–E) Xenium dataset quality control metrics, including (B) ratios of high‐to‐low quality transcript reads, (C) transcript localization, (D) transcript spatial density, and (E) counts based on cell segmentation (n = 4 each, ^**^ *p*<0.01). (F,G) Spatial transcript mapping back onto PCL‐implanted tissue showing (F) transcript density across PCL cross‐sections and (G) single‐cell transcript expression. (H) Representative histological‐based Masson's trichrome staining of PCL cross‐sections (scale bars = 1000 µm, inset scale bar = 250 µm).

Spatial transcriptomics has been largely applied to native unmodified tissues, with limited studies exploring its use in tissues implanted with synthetic materials. To validate that the presence of subcutaneously implanted biomaterials did not impact transcript capture or read quality, we conducted a quality control analysis of Xenium‐generated data from both control mouse skin tissue and mouse skin tissue implanted with an electrospun PCL scaffold (male C57B/L6 mice, 9–10 weeks old, dorsal subcutaneous implant for 14 days) (Figure 1A). High‐quality to low‐quality transcript ratios were highly comparable between the two conditions, with skin tissue yielding 85.2% and 14.8% high‐ and low‐quality transcripts, respectively, while scaffold‐implanted samples showed nearly identical ratios of 85.4% and 14.6% (Figure 1B).

Using complementary quality metrics, including percentages of overall transcripts detected within cells and detected cells without transcripts, provided insights into the accuracy of transcript‐to‐cell mapping (Figure 1C). No significant differences in transcripts detected in cells nor cells without transcripts were observed between the two groups, suggesting that transcript localization at single cell resolution was not compromised. Additional quality metrics, such as transcripts per µm^2^ and cells per mm^2^, showed significant differences in PCL samples, measured to have a 2.02‐fold increase compared to control skin tissue (Figure 1D,E). Increased cell density detected in PCL samples was expected as a potential result of enhanced cell recruitment, highlighting that accurate cell segmentation was also not compromised by scaffold implantation.

Spatial mapping of all captured transcripts revealed that most of the gene expression induced by scaffold implantation was captured in the surrounding capsule and body of PCL scaffolds (Figure 1F). Integration of cell segmentation data further allowed for visualization of individual cells and identified those with the highest overall transcription expression to be in the surrounding capsule and body of PCL scaffolds (Figure 1G). Increased transcriptional activity, likely by elevated cell density, was corroborated by Masson's trichrome staining (Figure 1H). In contrast, control tissue samples exhibited minimal gene expression and low cell density in the subcutaneous layer (Figure S1), suggesting that both patterns were driven by responses to scaffold implantation.

Collectively, the presence of PCL scaffolds did not interfere with transcript capture nor impact output data quality or visualization. For broader application of spatial transcriptomics in biomaterial evaluation, this provides promising evidence suggesting that high‐dimensional transcriptomic data capture could remain viable in the presence of scaffolds with similarly diverse physicochemical features like hydrophobicity and nanotopography, which are not native to biological tissue. Future study of a broader range of biomaterials with diverse compositions, structures, and surface chemistries could generate critical insights to support widespread adoption in the field. Current studies remain limited, with only two reported applications having investigated silicon‐based electrode arrays for neural stimulation [12] and electrospun PLGA scaffolds in wound healing models [11]. Establishing a quality control framework represents a crucial first step within a broader workflow to ensure data integrity for accurate downstream analysis.

### 3.2 Global Transcriptomic Profiling

**FIGURE 2:**
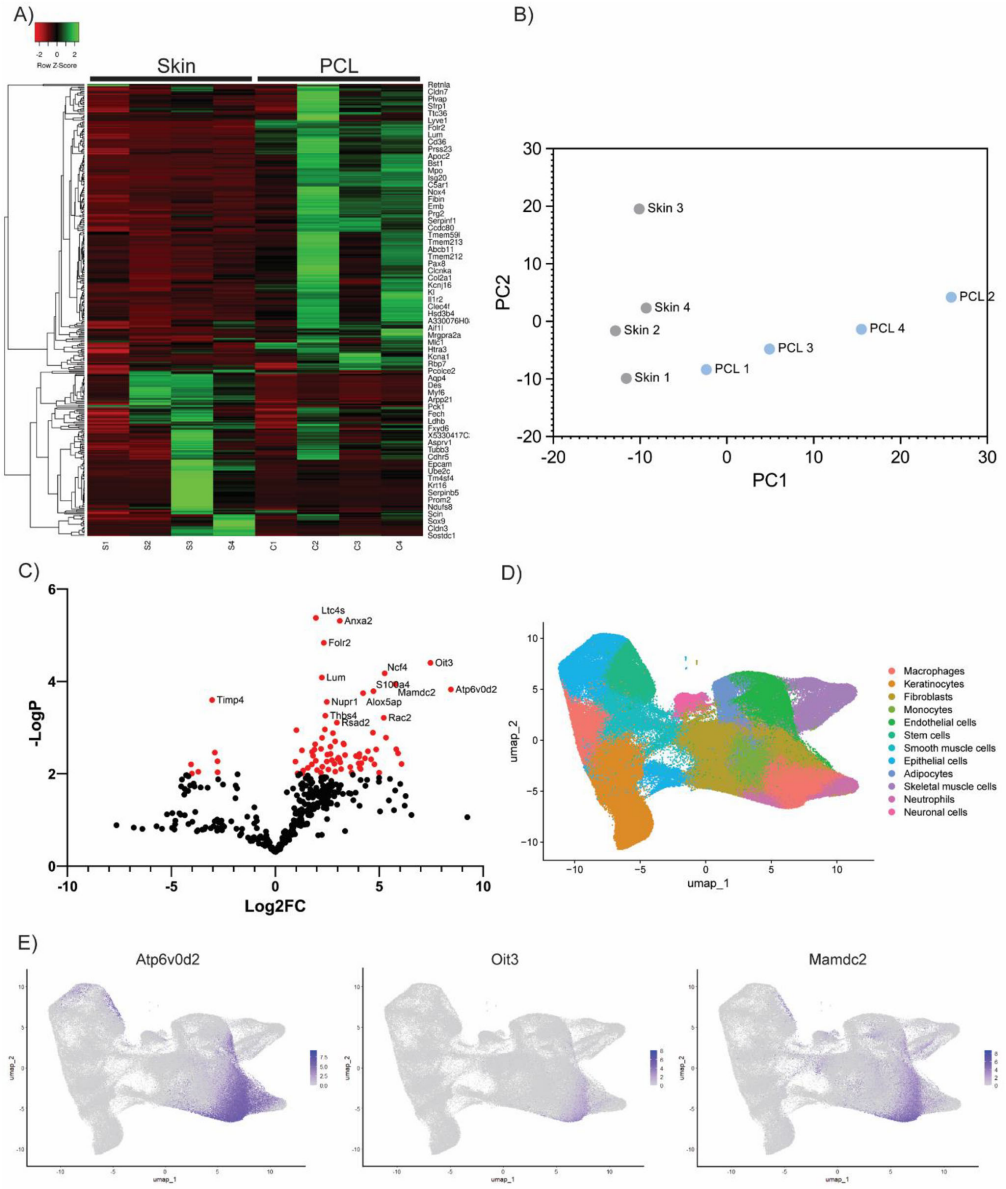
Computational and biological benchmarking of spatial datasets. (A) Hierarchical clustered heatmap comparing transcriptomic profiles of PCL‐implanted tissue and control skin. (B) Principal component analysis (PCA) with distinct separation of PCL and control skin samples. (C) Volcano plot of differential gene expression between PCL and skin tissue, significantly upregulated genes (log_2_FC ≥ 1, –log_10_p ≥ 2) highlighted in red. (D) UMAP visualization of integrated skin and PCL samples using the top 15 principal components. (E) Feature plots of Mamdc2, Oit3, and Atp6v0d2 localized within macrophage clusters.

Validation of dataset quality enabled assessment of its accuracy against established computational and biological standards. Global transcript expression profiles and underlying high‐dimensional datasets of all samples were evaluated for compatibility with traditional bioinformatics workflows. Hierarchical clustered heatmapping revealed distinct gene expression patterns between control skin and PCL tissue (Figure 2A). Complementary gene expression patterns across multiple clusters were observed, where genes upregulated in skin tissue were downregulated in PCL tissue, and vice versa. This transcriptional heterogeneity was verified using principal component analysis (PCA), which identified 15 principal components accounting for a variance of ≥ 67% (Figure S2). Distinct clusters between skin and PCL samples were formed primarily along PC1, accounting for 53% of variance (Figure 2B). This suggested that PCL implantation induced distinct transcriptional changes as expected, confirming that standard bioinformatics tools can effectively capture broad shifts in gene expression within scaffold‐implanted tissue. Future studies using this workflow could test whether it can detect subtle differences in gene expression which reflect how cells respond to biomaterials with slight design modifications. This could enable a more data‐driven approach to fine‐tune material designs for better tissue responses.

Leveraging the granularity of transcriptomic data enables a more detailed exploration of tissue responses at the genetic level, which can be used to broadly inform underlying biological processes and cell function. By using volcano plots to visualize differential gene expression comparing PCL‐implanted tissue relative to native skin, we identified significantly upregulated genes within the dataset (log_2_FC of ≥ 1 and a ‐log10 *p*‐value of ≥ 2). This approach revealed 40 genes (red dots), with Mamdc2, Oit3, and Atp6v0d2 among the most highly upregulated (Figure 2C). All three genes have been biologically validated to play key roles in processes responding to scaffold implantation. Mamdc2 has been implicated in modulating cytokine production and immune signaling in epithelial cells during injury [19]. Oit3 has been shown to regulate extracellular matrix production and fibrotic responses in tissue repair [20, 21], while Atp6v0d2 has been linked to regulating macrophage activation and immune modulation [22].

Further exploring how these gene expression changes manifest within distinct cell populations provided deeper insights into the functional roles of individual cells within the surrounding scaffold‐tissue microenvironment. To identify and visualize different cell types present in the tissue samples, we combined the transcriptomic data from both skin and PCL‐implanted tissues and used an unsupervised clustering approach to group cells based on their gene expression patterns. Using a visualization technique called UMAP, which reduces multi‐dimensional data into a two‐dimensional plot, we found 12 distinct clusters of cells (a single cell represented as an individual dot on UMAP) with unique transcriptional profiles that existed within the combined PCL and skin samples (Figure S3). These clusters corresponded to a range of cell types, including macrophages, keratinocytes, fibroblasts, monocytes, stem cells, smooth muscle cells, epithelial cells, adipocytes, skeletal muscle cells, neutrophils, and neuronal cells (Figure 2D). To understand which of these cell clusters were expressing the key upregulated genes, we then mapped the expression of Mamdc2, Oit3, and Atp6v0d2 onto this UMAP plot. This revealed that these genes were primarily active in clusters identified as macrophages (Figure 2E), suggesting that macrophages are likely major contributors to the transcriptional and potentially functional remodeling responses following PCL scaffold implantation.

This analysis aligns well with established biological knowledge, as macrophages are known master regulators of foreign body inflammation. Mamdc3, Oit3, and Atp6v0d2 further highlight a transcriptional response consistent with inflammation, fibrosis, and tissue remodeling processes. These validations support the accuracy of the data against hallmark biological processes during the foreign body response, demonstrating that transcriptomic datasets of scaffold‐implanted tissue are compatible with traditional single‐cell bioinformatics analyses.

### 3.3 Spatial Tissue Integration of Transcriptomic Clusters

**FIGURE 3:**
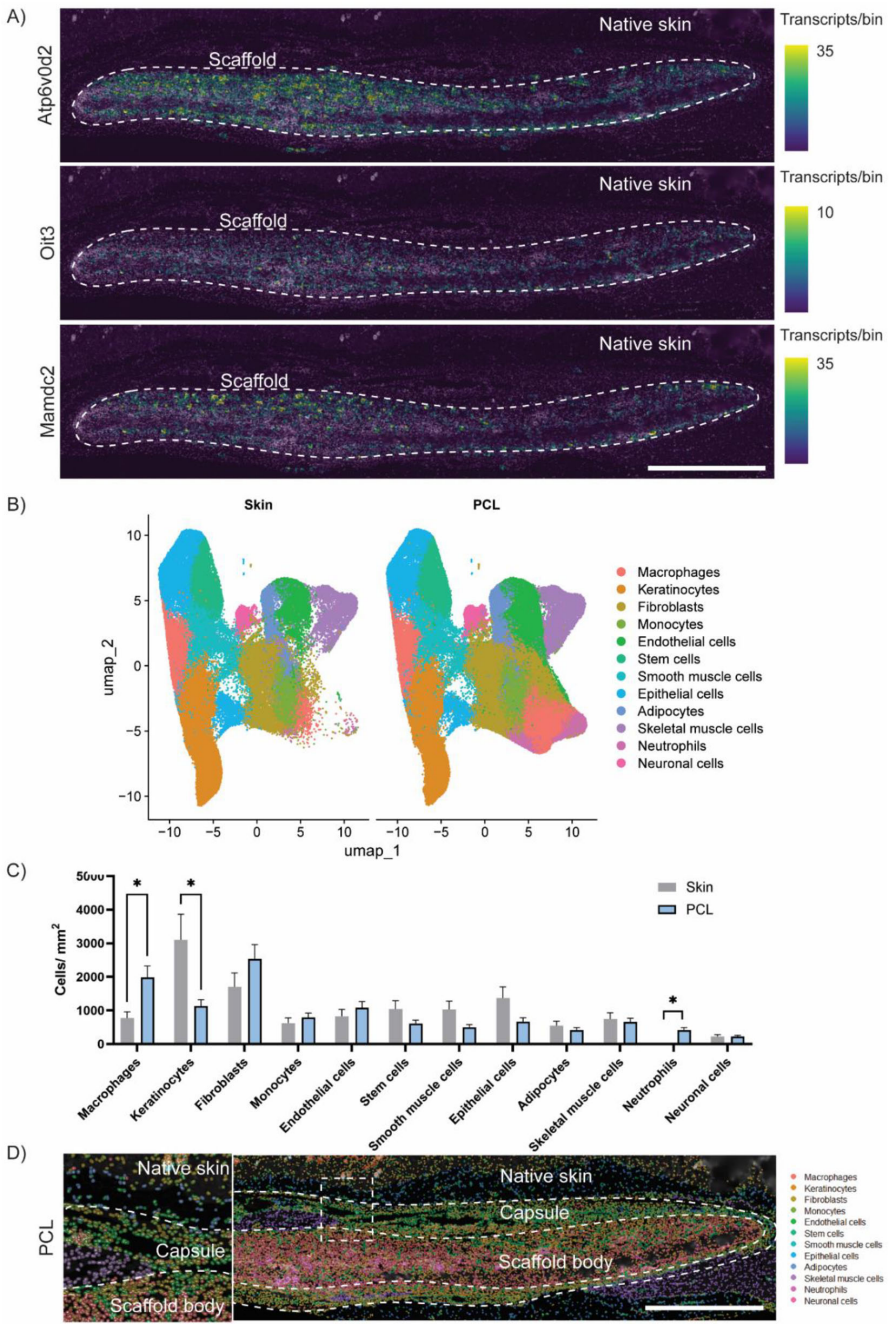
Spatial mapping of gene expression and transcriptionally defined cell clusters. (A) Spatial transcripts of Mamdc2, Oit3, and Atp6v0d2 mapped onto PCL‐implanted tissue. (scale bar = 1000 µm) (B) separated UMAPs of skin and PCL cell phenotype subclusters. (C) Quantification of cell phenotype subcluster frequency between skin and PCL (n = 4 each, ^*^ *p*<0.05). (D) Spatial mapping of transcriptionally defined cell clusters back onto respective tissue samples showing localization in distinct anatomical regions of native skin, capsule, and scaffold body (scale bar = 1000 µm).

Expanding upon traditional bioinformatics frameworks, integration of spatial data could allow for a more nuanced understanding of material responses, as biological interactions can vary across the surface of a biomaterial, influenced by both the inherent material properties and the heterogeneous complexity of the tissue it contacts. Spatially mapping Mamdc2, Oit3, and Atp6v0d2 transcript expression back onto PCL tissue highlighted this heterogeneity (Figure 3A). Transcript density was notably localized to the scaffold capsule and body, with a greater concentration observed on the surfaces facing the epidermis, rather than the subcutaneous layer. This spatial localization of transcripts was orthogonally validated using immunohistochemistry, confirming localization of the protein encoded by the mapped transcripts within the scaffold‐capsule interface (Figure S4). Furthermore, gene expression was not uniform along the length of the scaffold, instead concentrating at singular points at the scaffold surface. Since earlier analysis showed that these genes were predominantly expressed in macrophages, this suggests that macrophages recruited to the epidermis‐facing surface of the implant were comprising a major component of these tissue responses to PCL scaffolds. While this was largely expected, the added spatial resolution provided valuable granularity, precisely identifying where macrophage‐scaffold interactions occurred and the degree to which these interactions vary across different regions of the scaffold surface. While macrophage activity can only be inferred through this approach, we hypothesized that cell clusters identified from the integrated UMAP and mapped back onto PCL tissue would validate and provide direct visualization of all cell‐scaffold interactions.

To accomplish this, we first segregated the integrated UMAP by sample type, generating UMAPs for skin and PCL samples (Figure 3B). This revealed that while overall cluster structure remained consistent between samples, expected changes in the abundance of cell populations like macrophages were observed, indicated by fewer individual dots representing those cells on the UMAP (bottom right, pink). Cell populations that remained unchanged between PCL and skin samples were initially presumed to be cells which were tissue‐resident/already present in the skin prior to scaffold implantation. In contrast, changes in populations, such as macrophages, suggested a recruitment or activation response triggered by the implantation of PCL scaffolds. Quantifying these changes by calculating the frequency of cells within each cluster revealed significant increases of 2.6‐ and 26‐fold in macrophages and neutrophils, and a 2.7‐fold decrease in the keratinocytes when comparing PCL to skin samples (Figure 3C). To visualize these cell‐scaffold interactions, PCL cell clusters were then mapped back onto the tissue (Figure 3D), revealing a highly organized spatial distribution with clusters localized to distinct regions within the scaffold body, capsule, or native skin (Figure S5).

Additionally, this full transcriptomic analysis was applied to an independent Day 3 PCL implant dataset to further examine the robustness of our pipeline in capturing early immune and tissue responses (Figure S6). PCA revealed a clear separation between Day 3 PCL and skin control samples, indicating extensive transcriptional disruption consistent with the acute response immediately following scaffold implantation. Volcano plot analysis identified 2610528A11Rik, Cstdc4, and Cd5l as the most strongly upregulated genes. UMAP clustering and cluster‐frequency analysis further showed that this early response was characterized by a marked increase in monocytes and macrophages and a reduction in epithelial cells—expected features of the initial inflammatory phase after implantation. FeaturePlot mapping of the top genes demonstrated that 2610528A11Rik, a keratinocyte‐associated transcript implicated in epithelial activation, and Cstdc4, a cystatin domain–containing protease inhibitor linked to epithelial barrier maintenance, were both highly expressed in epithelial and keratinocyte clusters, reflecting epidermal activation and remodeling at the wound site. In contrast, Cd5l, a macrophage‐secreted scavenger receptor–associated molecule involved in lipid metabolism and inflammatory regulation, was enriched in macrophage clusters, consistent with early macrophage infiltration and activation around the scaffold. These findings capture the acute epithelial–immune interplay characteristic of the early implantation phase and more broadly highlight the pipeline's capacity to sensitively resolve biologically meaningful transcriptional dynamics at early timepoints. This reinforces the utility of the workflow as a high‐resolution tool for profiling evolving cellular states across multiple timepoints and implant contexts.

These findings underscore the value of integrating spatial data with traditional bioinformatics approaches to refine our understanding of biomaterial‐host interactions. Mapping transcriptionally distinct cell clusters back onto the tissue provided a more precise visualization of how scaffold‐responsive cells, particularly macrophages, localize within and around the implant. This spatial resolution was validated against expected immune responses, such as increases in macrophage and neutrophil clusters, but also aligns with anticipated tissue changes, including decreased keratinocytes liking arising from injury to the skin during implantation. The ability to simultaneously profile multiple cell types and visualize their organization within a single cross‐section enhances this capacity, revealing coordinated cell interactions that may be overlooked using traditional histology. Capturing multiple processes, such as immune and tissue remodeling in a highly spatially resolved manner could be widely beneficial in linking distinct cell behaviors to material properties.

### 3.4 Macrophage Subclustering

**FIGURE 4:**
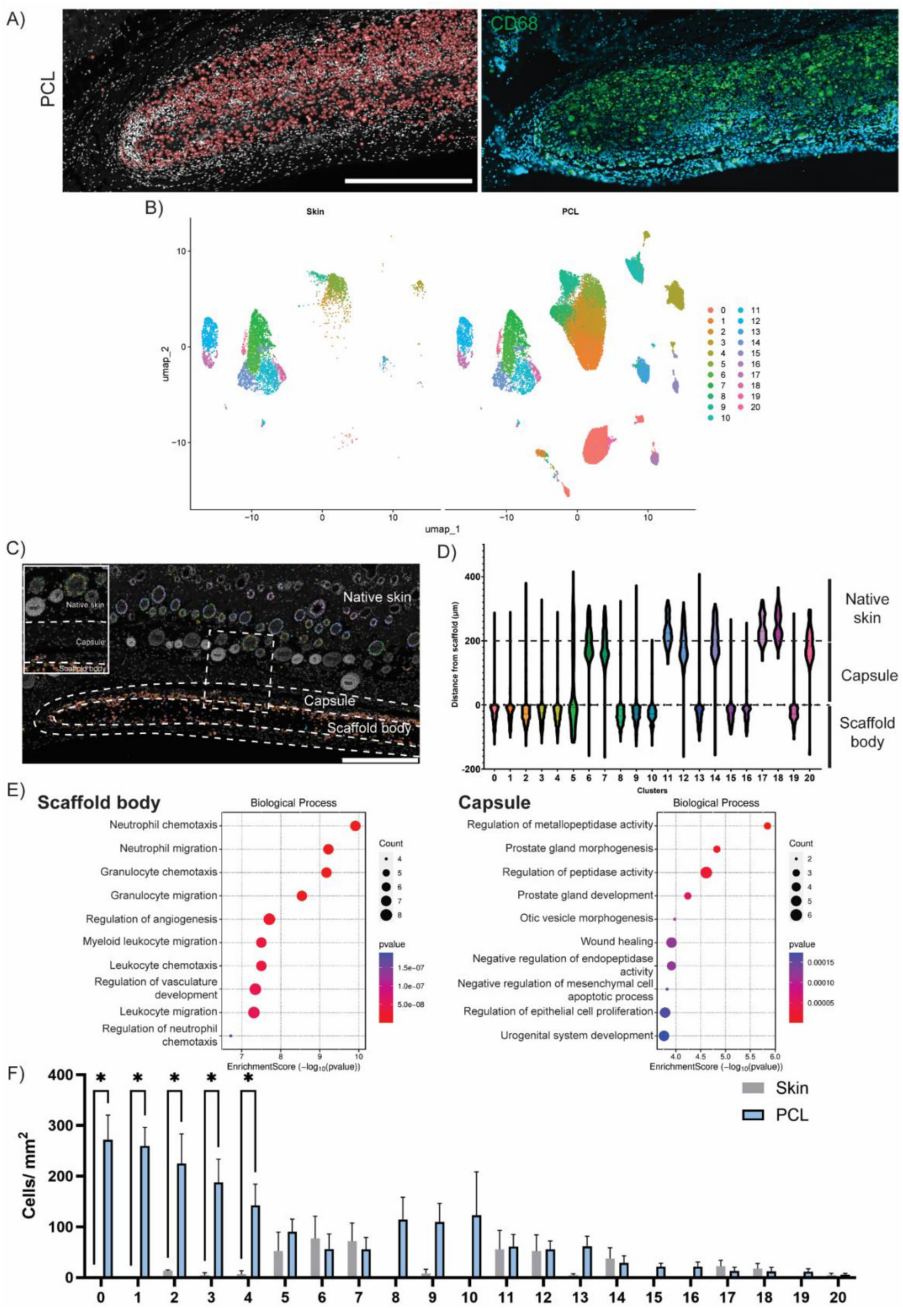
Macrophage subclustering of phenotypically and spatially distinct states (A) Orthogonal validation of macrophage subcluster phenotyping using CD68 immunohistochemical staining for CD68, confirming spatial overlap (scale bar = 500 µm). (B) UMAP visualization of macrophage subclusters between skin and PCL tissue. (C) Spatial mapping of macrophage subclusters onto PCL‐implanted tissue (scale bar = 500 µm). (D) Violin plots of localization analysis quantifying the spatial distribution of each macrophage subcluster within each of the 3 regions. (E) Gene ontology enrichment analysis of scaffold‐ and capsule‐localized subclusters. (F) Cluster frequency analysis quantifying significantly upregulated macrophage subcluster populations between skin and PCL tissue (*n* = 4 each, ^*^ *p* < 0.05).

To explore this further, we performed subclustering of the macrophage population, grouping them into finer subsets based on distinct gene expression profiles. This analysis was motivated by their observed abundance in PCL tissue and their well‐established role in mediating foreign body responses. The accuracy of macrophage clustering was first orthogonally validated using immunohistochemistry. CD68 staining showed high alignment with spatial mapping of the macrophage cluster, which appeared limited to either the scaffold body or capsule (Figure 4A), supporting the accuracy of clustering phenotyping. Subclustering was then performed by again selecting the first 15 principal components for UMAP visualization, but this time focusing exclusively on the macrophage cluster within skin and PCL samples (Figure 4B). This approach identified 21 transcriptionally distinct sub‐populations (Figure S7), which, when mapped back onto PCL tissue, successfully revealed the original macrophage cluster as a composite of these finer subsets (Figure 4C).

Using a custom‐designed computational tool for spatial analysis on this subclustered map, we quantified the distance of each cell within the subclusters relative to the scaffold surface, distinguishing cells within the scaffold body from those in the capsule. Amongst the 21 macrophage subclusters, 13 were localized in the scaffold body, 5 in the capsule, and 3 subclusters were found in the native skin (Figure 4D). For the macrophage subclusters located within the scaffold body and capsule, we then performed gene ontology enrichment analysis, a method that uses curated biological databases to classify groups of genes into known cell pathways and functions. The genes selected from each subcluster were defined by genes with a log_2_FC > 0.25 and at least 50% expression within the subcluster. Macrophages within the scaffold body showed the highest enrichment for immune cell recruitment processes, such as neutrophil, granulocyte, and leukocyte chemotaxis, while macrophages in the capsule were enriched for processes associated with fibrotic capsule formation, including regulation of metallopeptidase activity and peptidase activity and wound healing (Figure 4E). This suggested that macrophages exhibited distinct functions based on their location. Within the capsule, macrophages were driving capsule organization, while macrophages within the scaffold body were regulating immune cell recruitment. Further quantification of subcluster frequencies revealed that the most pronounced changes in macrophage sub‐populations occurred within subclusters 0–4, all located in the scaffold body (Figure 4F), indicating them as the key macrophage responders to scaffold implantation. Complementary histology of the top marker genes for each subcluster broadly supported the regional accuracy of the spatial mapping of these subclusters (Figure S8).

This subcluster‐level analysis was valuable in highlighting which macrophage sub‐populations were upregulated, suggesting enhanced involvement in response to PCL scaffold implantation. Combined with our localization analysis tool, these five macrophage subclusters were predominantly located within the scaffold body. This could suggest that the internal biomaterial microenvironment is actively shaping macrophage phenotypes through selective interactions and material‐specific cues. It should also be noted that these macrophage subclusters represent a snapshot in time, capturing transitioning phenotypes as transcriptionally distinct states and further underscoring the dynamic heterogeneity of the comprehensive macrophage response. Capturing this heterogeneity demonstrates the ability to infer significantly deeper than a single CD68 stain, allowing for more informed hypotheses about underlying cellular mechanisms and spatially distinct behaviors.

### 3.5 Fibroblast Subclustering

This approach can be theoretically further applied to all remaining cell clusters within the integrated PCL and skin dataset, providing a comprehensive view of the transcriptional landscape underlying the foreign body response. While the entire analysis was not conducted here, we aimed to demonstrate the limitations of integrated clustering analysis, which may capture total cell population shifts but overlook subtle changes at the subcluster level. Subcluster analysis, as observed with macrophages, allows detection of shifts in specific sub‐phenotypes that may be masked at the integrated level due to opposing changes in other subpopulations. For example, fibroblasts showed no significant changes at the integrated analysis level (Figure 3C), their well‐established role in scaffold remodeling and foreign body fibrosis made it essential to analyze them, highlighting the utility of subcluster analysis within the overall workflow.

**FIGURE 5:**
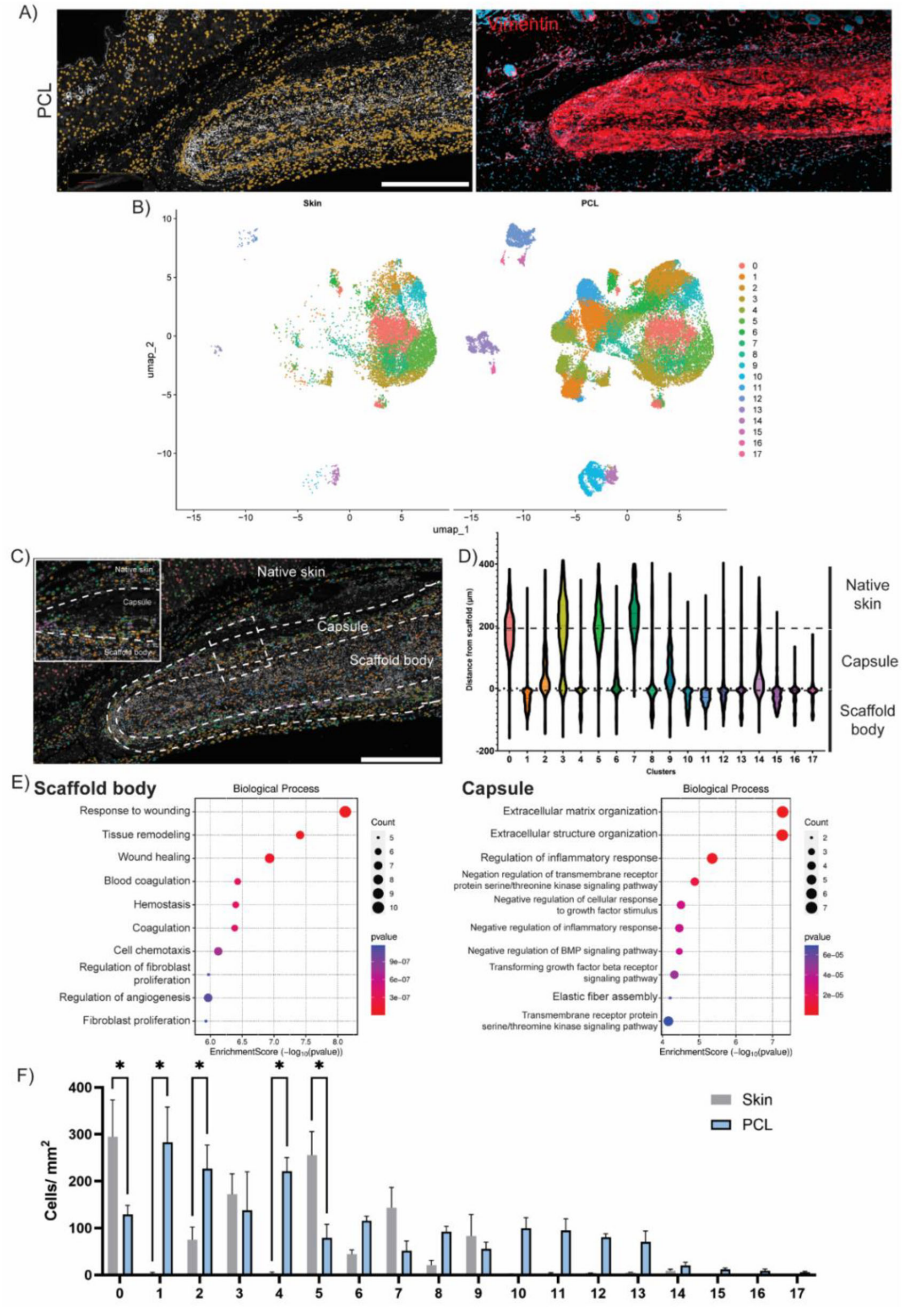
Fibroblast subclustering of phenotypically and spatially distinct states (A) Orthogonal validation of fibroblast subcluster phenotyping using immunohistochemical staining for Vimentin, confirming spatial overlap (scale bar = 500 µm). (B) UMAP visualization of fibroblast subclusters between skin and PCL tissue. (C) Spatial mapping of fibroblast subclusters onto PCL‐implanted tissue (scale bar = 500 µm). (D) Violin plots of localization analysis quantifying the spatial distribution of each fibroblast subcluster within each of the 3 regions. (E) Gene ontology enrichment analysis of scaffold‐ and capsule‐localized subclusters. (F) Cluster frequency analysis quantifying significantly regulated fibroblast subcluster populations between skin and PCL tissue (n = 4 each, ^*^ *p*<0.05).

Similarly, the accuracy of integrated clustering was first orthogonally validated using immunohistochemistry. Traditional fibroblast marker, vimentin staining, showed high alignment with spatial mapping of the fibroblast cluster, which appeared in all scaffold body, capsule and skin regions (Figure 5A). Subclustering of this population (Figure S9) identified 18 transcriptionally distinct sub‐populations (Figure 5B), which, when mapped back onto PCL tissue, similarly revealed the original cluster as a composite of these subclusters (Figure 5C). Localization analysis of cell distance to scaffold revealed that amongst the 18 fibroblast subclusters, 11 were localized in the scaffold body, 4 in the capsule, and 3 subclusters were found in the native skin (Figure 5D). Using only scaffold body and capsule as spatial context, ontology analysis revealed that fibroblasts within the scaffold body were significantly enriched for wound healing processes such as tissue remodeling, response to wounding, haemostasis, and chemotaxis, while fibroblasts within the capsule were enriched for processes associated with fibrotic capsule formation, including extracellular matrix organization, structural organization, and immune responses (Figure 5E). This suggested that scaffold fibroblasts were involved in the initial stages of tissue repair and inflammation, while capsule fibroblasts were more associated with stages of fibrotic encapsulation, highlighting their dynamic and spatial‐dependent behaviors.

Cluster frequency analysis of the subclusters identified significant differences in 5 sub‐populations, mixed between up‐ and down‐regulated when comparing PCL to skin samples (Figure 5F). This explains why these changes were not detected at the integrated level, as the magnitude of upregulations and downregulations likely offset each other. It also suggests that scaffold remodeling involves the dynamic regulation of fibroblast phenotypes, with upregulated clusters (1, 2, and 4) predominantly located in the scaffold body and being associated with wound healing processes, and downregulated clusters (0 and 5) localized mainly to the scaffold capsule, contributing to the organization of extracellular matrix and structure. This would be consistent with our macrophage findings, which indicate that macrophages within the scaffold body showed evidence of heightened immune cell recruitment, further emphasizing the role of cells within the scaffold body in orchestrating scaffold remodeling.

Complementary histology of the top marker genes for each of the significantly upregulated fibroblast subclusters broadly recapitulated the spatial transcriptomic mapping observed, validating regional subcluster assignments while highlighting expected differences between transcript and protein localization (subcluster 2) due likely to post‐transcriptional regulation and/or protein trafficking (Figure S10). These observations highlighted that complementary histology broadly validates subclusters at the regional level, but primarily serves to provide deeper biological insight into cell function and behavior, and should be interpreted alongside spatial transcriptomic mapping.

By revealing the dynamic regulation of fibroblast and macrophage subpopulations, where distinct clusters are differentially engaged in wound healing, extracellular matrix organization, and immune modulation, this approach highlights the spatial heterogeneity underlying scaffold remodeling. Ultimately, these insights underscore the value of a spatially resolved framework for advancing our understanding of biomaterial–tissue interactions.

### 3.6 Spatial Cluster Relationships

Spatial data can be further leveraged to provide an even deeper, more mechanistic understanding of biomaterial–tissue interactions, capturing the cellular context and spatial organization of responding cell types/upregulated subclusters. To explore these interactions, we developed a custom colocalization analysis tool that integrates spatial coordinates of each cell to infer physical relationships between subclusters. Applying this tool to our subcluster analysis of macrophages and fibroblasts allowed us to determine how spatial proximity may contribute to their individual and coordinated responses to PCL implantation. Specifically, we analyzed the degree of colocalization between all upregulated PCL subclusters within macrophages (0, 1, 2, 3, and 4) and fibroblasts (1, 2, and 4) (gene marker found in Table S1), generating a pairwise matrix of interaction patterns across all subcluster combinations.

**FIGURE 6:**
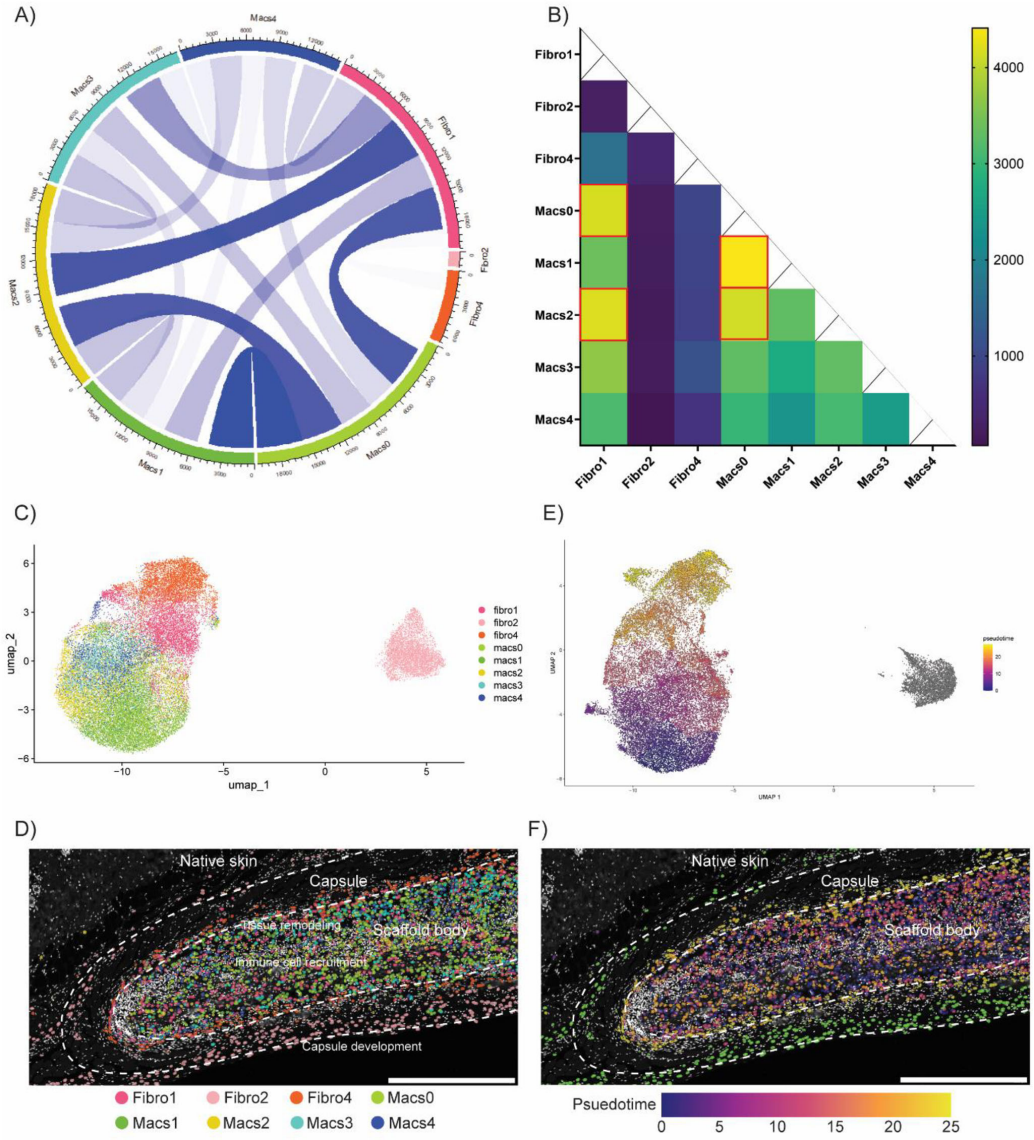
Integration of spatial colocalization and phenotypic similarity reveals transcriptional continuum and spatially oriented cell‐state organization. (A) Chord diagram visualization of upregulated macrophage and fibroblast subcluster interaction patterns using colocalization‐based interactions (large width and darker color indicate strength of interaction). (B) Heat map quantification of subcluster colocalization of specific pairwise relationships indicated the strongest subcluster interactions (red boxes). (C) UMAP embedding of upregulated macrophage and fibroblast subclusters. (D) Spatial mapping of UMAP clusters onto PCL tissue (scale bar = 500 µm). (E) Application of trajectory inference using Monocle 3 using pseudotime progression from early (purple) to late (yellow) stage along a transcriptional continuum within the main UMAP body; divergent pseudotime trajectory (green). (F) Spatial mapping of pseudotime trajectory onto PCL tissue (scale bar = 500 µm).

To quantify cell–cell interactions inferred from spatial proximity, we calculated the number of neighboring cells between each pair of subclusters as a proxy for physical interaction. Visualization of subcluster interactions using a chord diagram, which depicted the strength of interactions between subclusters, revealed that the strongest interactions occurred between macrophage subclusters themselves or between macrophages and fibroblasts, whereas interactions between fibroblast subclusters were minimal or absent (Figure 6A). A heat map detailing the strength of these interactions provided a quantitative view across all individual subcluster pairs. Among macrophage‐macrophage subcluster interactions, subcluster 0 (Macs0) showed the strongest interactions with subclusters 1 and 2 (Macs1 and Macs2). Amongst macrophage–fibroblast interactions, fibroblast subcluster 1 (Fibro1) showed the highest interaction with macrophage subclusters 0 and 2 (Macs 0 and Macs 2) (Figure 6B). Consistent with our visualization, fibroblast‐fibroblast interactions were minimal. Both remaining fibroblast subclusters, 2 and 4 (Fibro2 and Fibro4), showed little to no colocalization with any of the subclusters.

Using this spatial proximity to reveal key interaction patterns, we then sought to assess phenotypic similarity between these macrophage and fibroblast subclusters. To do this, we performed UMAP embedding of the macrophage and fibroblast subclusters to examine how they relate in transcriptional space. This UMAP revealed two tightly clustered groups, one comprising all macrophage subclusters and most fibroblast subclusters, and one consisting of fibroblast subcluster 2 (Fibro2) (Figure 6C). Mapping these subclusters back onto PCL scaffolds revealed a strong spatial correlation, with cells from the main cluster body primarily localized within the scaffold body and/or at the scaffold–capsule interface, and the isolated fibro2 subcluster predominantly located within the surrounding capsule and adjacent native skin regions (Figure 6D).

Integrating both this phenotypic data and the previous spatial colocalization data, we were able to infer potential interactions and transitions between macrophage and fibroblast subclusters. The close clustering of macrophage and fibroblast subclusters within the main UMAP group suggested shared or overlapping phenotypic profiles consistent with known macrophage and fibroblast interactions [23, 24]. To explore the relationship and potential transitions between these subpopulations, we applied Monocle 3, a trajectory inference tool that reconstructs dynamic patterns from gene expression profiles. The resulting trajectory, ordered along pseudotime, suggested a progression from macrophage subcluster 0 (Macs0—purple) at the bottom of the UMAP group toward fibroblast subcluster 4 (Fibro4 – yellow) at the top, passing through intermediate macrophage and fibroblast sub‐phenotypes (i.e., Macs1–4 and Fibro1) (Figure 6E). This progression reflects shared and intermediate transcriptional features among macrophage and fibroblast subclusters.

To further investigate the transcriptional relationship between macrophage and fibroblast subclusters, we defined two unsupervised gene panels corresponding to macrophage‐like and fibroblast‐like programs (Table S2). FeaturePlot analysis of these programs onto the combined macrophage–fibroblast UMAP revealed a clear transcriptional gradient with regions enriched for macrophage‐like genes (purple) localized toward the bottom of the UMAP, fibroblast‐like genes (orange) toward the top, and subclusters co‐expressing both programs positioned in‐between (Figure S11). This pattern is consistent with the previously inferred pseudotime trajectory, reflecting a continuum of shared phenotypic features oriented along a spatial gradient from the scaffold body toward the capsule. These results support the presence of overlapping transcriptional programs between macrophage and fibroblast subclusters within scaffold‐internal regions, highlighting potential cell state interactions without implying direct transdifferentiation.

When mapping this pseudotime analysis back onto the tissue, this inferred trajectory interestingly aligned with a clear spatial gradient (Figure 6F). Early and intermediate phenotypes along the trajectory (e.g., Macs0‐2 – purple/pink) were located primarily within the scaffold body and closely associated with one another, confirming our previous colocalization analysis. In contrast, fibroblast subcluster 4 (Fibro4, representing the terminal phenotype in the trajectory – orange/yellow), was found at the scaffold–capsule interface, also consistent with our previous colocalization analysis that showed its minimal association with other subclusters. These findings suggest the existence of a macrophage‐to‐fibroblast transcriptional continuum within the scaffold body, oriented along a directional gradient toward the scaffold–capsule interface, with a fibroblast subcluster representing a distinct terminal transcriptional state at the scaffold surface.

In contrast, fibroblast subcluster 2 (Fibro2—grey) stood out as both phenotypically and spatially distinct (Figure 6E). It showed minimal association with main UMAP body and did not interact with any macrophage or fibroblast subtypes in the colocalization analysis. When mapped onto PCL implants, Fibro2 (green) was localized mainly within the capsule and adjacent native skin (Figure 6F). This spatial segregation, combined with its unique transcriptional profile, suggests that this subpopulation may originate from tissue‐resident fibroblasts rather than being derived from macrophage subclusters associated with the scaffold response. This is further supported by Fibro2 marker genes, including Pi16, Dpt, and Mfap5 (Table S1), which are well‐established indicators of tissue‐resident fibroblasts [25].

Together, these findings highlight the power of integrating spatial transcriptomics with high‐resolution transcriptional clustering to uncover the cellular context and interactions driving biomaterial responses. By combining spatial proximity with phenotypic similarity, we were able to infer not only which cells are interacting, but also how those interactions evolve over space and time. This approach moves beyond static, morphology‐based histological assessments and enables a mechanistic understanding of how specific subpopulations are organized, regulated, and functionally specialized within the tissue microenvironment. For example, for macrophages localized within PCL, we previously showed them to be enriched for GO terms associated with chemotaxis, suggesting a role in sustained immune cell recruitment [26]. Our pseudotime and spatial analyses further revealed that these macrophages occupy a transcriptional continuum with fibroblast‐like phenotypes, consistent with previous reports of closely associated macrophage and fibroblast states at biomaterial interfaces. [23, 24]. These hybrids migrate toward the scaffold surface, which we previously showed are enriched for GO terms related to wound healing and chemotaxis, implicating them in orchestrating both inflammatory and fibrotic processes at the capsule interface [27]. In contrast, fibroblasts derived from the surrounding capsule or native skin were spatially distinct and were enriched for GO terms related to extracellular matrix organization, suggesting their involvement in structural remodeling and fibrotic capsule formation [28].

**FIGURE 7:**
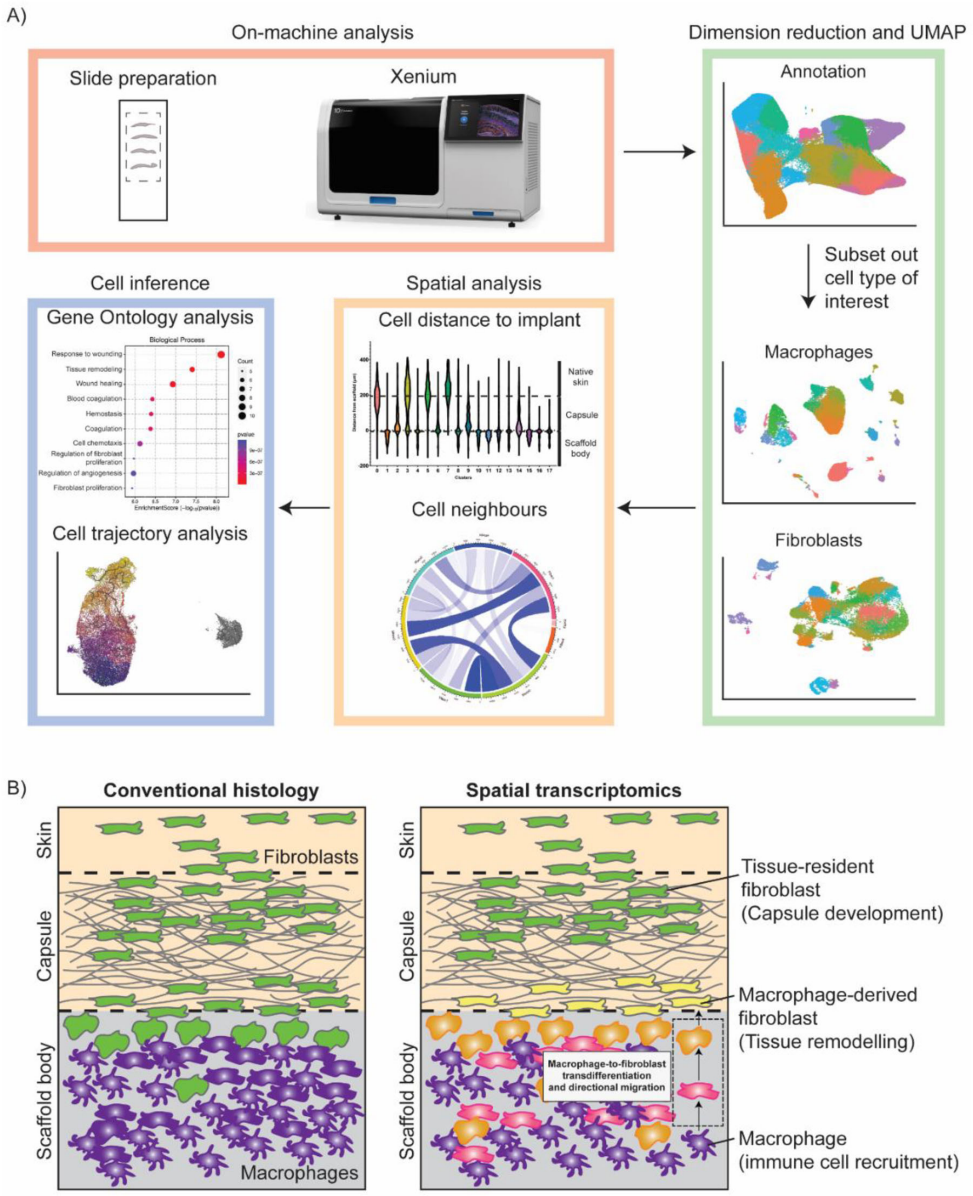
Schematic workflow of spatial transcriptomic analysis pipeline and proposed schematic summary of spatial and transcriptional dynamics during the foreign body response to PCL scaffold implantation, offering enhanced granularity beyond traditional histological assessment. (A) Slide preparation for Xenium on machine analysis is followed by standard dimension reduction and UMAP. Clusters were annotated, and subsets of cell types are selected for spatial analysis. Cell inference tools are then used to examine function and cell behavior. (B) Integrated spatial transcriptomics and high‐resolution clustering reveal coordinated cellular responses across the scaffold microenvironment. Macrophages localized within the scaffold body exhibit transcriptional profiles related to immune cell recruitment (GO: chemotaxis) and are inferred to undergo transdifferentiation toward fibroblast‐like states. These hybrid phenotypes migrate toward the scaffold surface and capsule interface, where they contribute to tissue remodeling (GO: wound healing and inflammatory signaling). Fibroblasts originating from the surrounding capsule or native skin remain spatially and transcriptionally distinct, contributing to capsule development (GO: extracellular matrix organization).

This spatially resolved, transcriptome‐driven approach to understanding scaffold remodeling offers a robust framework for uncovering the dynamic and spatially organized cellular interactions that drive biomaterial–tissue integration. Our study introduces a standardized, high‐resolution workflow for single‐cell spatial transcriptomics in biomaterials, leveraging the 10x Xenium platform. Key innovations include robust single‐cell subclustering of immune and stromal populations to resolve transcriptionally distinct subpopulations, spatial localization analyses relative to scaffold architecture, customized colocalization metrics to quantify interactions between defined subclusters, subcluster‐level gene ontology enrichment to link spatial organization with function, and shared transcriptional phenotypes oriented along directional spatial gradients, providing insight into potential cell‐state relationships to map directional cell state transitions. Together, these features form a reproducible, stepwise pipeline that can be applied broadly across diverse biomaterial contexts, providing a framework for future studies to capture cellular and spatial dynamics without redesigning analyses for each new system (Figure 7A). Importantly, the biological findings in this work primarily serve to demonstrate the utility and validity of this analytical framework. The proposed framework for applying spatial transcriptomics to in vivo biomaterial evaluation is both timely and necessary, given rapid and growing advances in spatial biology technologies. To date, the application of spatial transcriptomics within the biomaterials field has been limited, with existing studies largely confined to single material types or narrowly defined questions and outcomes [11, 12]. The absence of standardized workflows will only continue to hinder broader adoption of these platforms. Offering a systematic and universal approach for assessing the spatial organization, function, and mechanistic roles of specific cell populations in biomaterial responses, this workflow lays the groundwork for more rigorous, reproducible, and comprehensive evaluation of biomaterials across diverse tissue contexts (Figure 7B).

## 4 Conclusion

The workflow presented in this study provides a multidimensional view of the foreign body and scaffold remodeling responses, revealing how spatial location, phenotypic plasticity, and function are tightly coupled. Such insight is not achievable through traditional histology alone and offers a transformative toolset for better biologically‐informed biomaterial design and optimization. Continued advances in the use of spatial biology may enable the development of biomaterials that not only minimize adverse responses and improve biocompatibility but also actively guide desirable tissue outcomes by precisely modulating the local cellular microenvironment.

## Author Contributions

**Alex H.P. Chan**: data curation, formal analysis, visualization, investigation, writing – original draft. **Yunfei Hu**: data curation, formal analysis. **Billie Pardavi**: formal analysis. **Xueying Xu**: investigation, data curation. **Angus J. Grant**: investigation, data curation. **Steven G. Wise**: writing – review and editing, supervision, and funding acquisition. **Lipin Loo**: writing – review and editing, formal analysis. **Richard P. Tan**: conceptualization, supervision, project administration, writing – original draft, funding acquisition.

## Funding

This work was supported by NSW Health in the form of an NSW Cardiovascular Early‐Mid Career Researcher Grant (S.G.W & R.P.T.). S.G.W receives funding as a National Heart Foundation Future Leader Fellow. R.P.T receives funding as an Australian Research Council Early Career Industry Fellow. L.L. receives funding from the National Health and Medical Research Council (APP2019164) and the University of Sydney (Dr. John and Anne Chong Fellowship).

## Conflicts of Interest

The authors declare no conflicts of interest.

## Supporting information




**Supporting file**: advs74449‐sup‐0001‐SuppMat.docx.

## Data Availability

The data supporting the findings of this study are available from the corresponding author upon reasonable request. Custom R code used for data analysis is available at https://github.com/ahpchan/STimplant.
