# Supplementary material for: Developing a Single‐Cell Spatial Transcriptomics Workflow for In Vivo Evaluation of Implanted Biomaterials
Source: Adv Sci (Weinh). 2026 Feb 15;13(24):e13242. doi: 10.1002/advs.202513242 (PMC13116268; doi:10.1002/advs.202513242)
Supplement: Supplementary file 1 — Supporting file: advs74449‐sup‐0001‐SuppMat.docx. [file ADVS-13-e13242-s001.docx]

# Supplemental Information


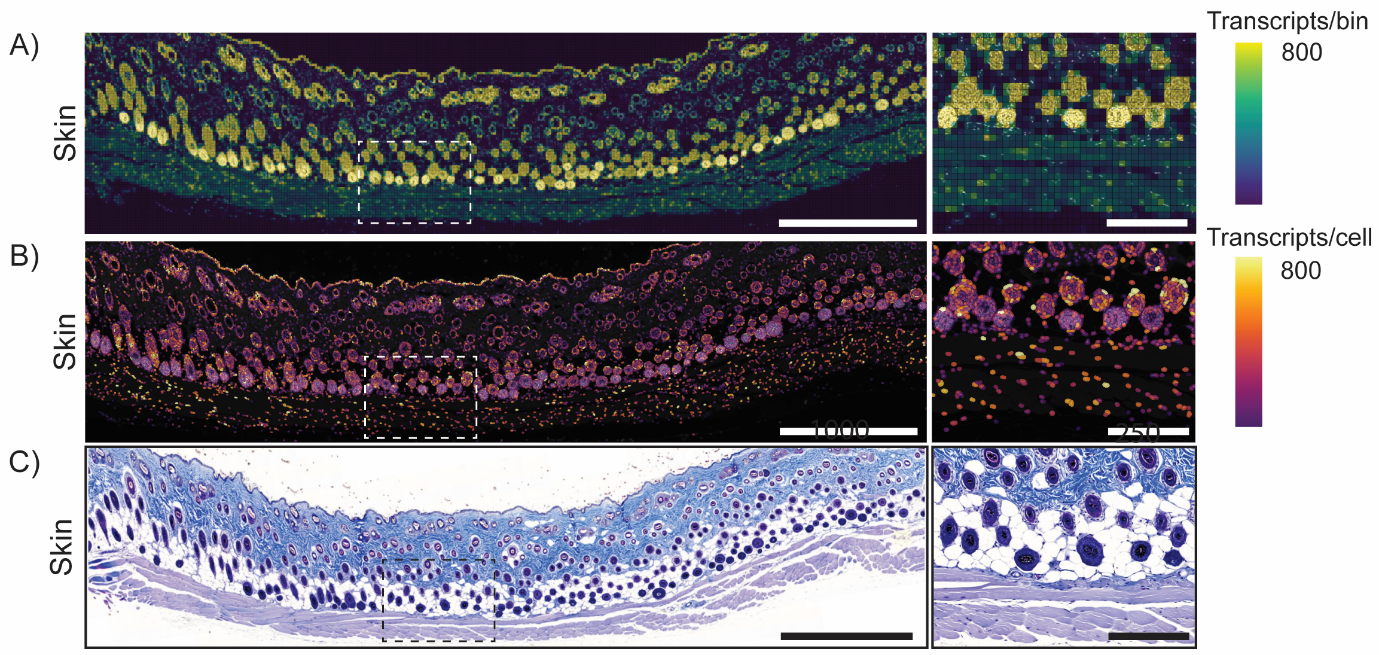


**Supp. Figure 1: Representative images of control skin tissue.** A-B) Spatial transcript mapping back onto control skin tissue showing (A) transcript density and (B) single-cell transcript expression. C) Representative histological-based Masson’s trichrome staining of PCL cross-sections (scale bars = 1000 µm, inset scale bar = 250 µm).


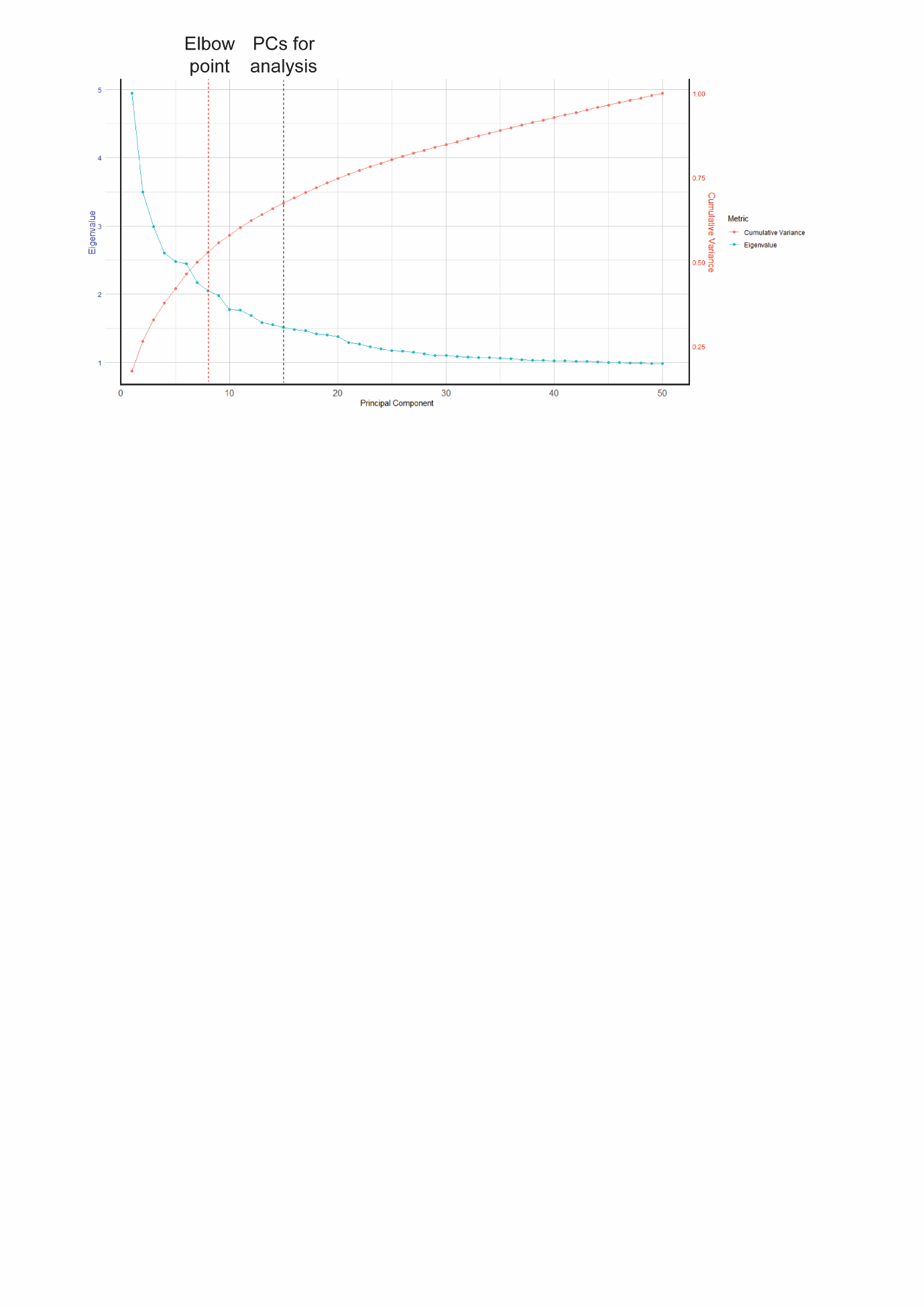


**Supp. Figure 2: Eigen value vs. principal component.** Elbow point is indicated by red dotted line. PCs used for analysis indicated by black dotted line.


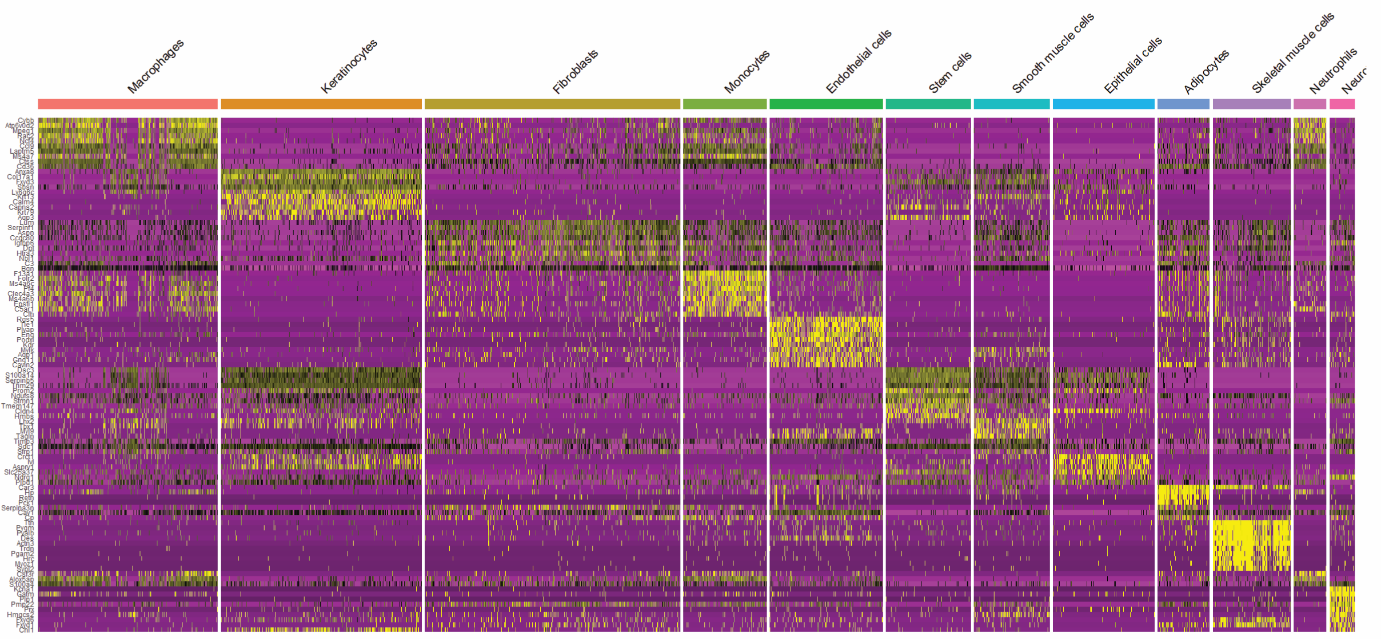


**Supp. Figure 3: Heatmap of top 10 upregulated genes for each global cluster of annotated cell type.**

**
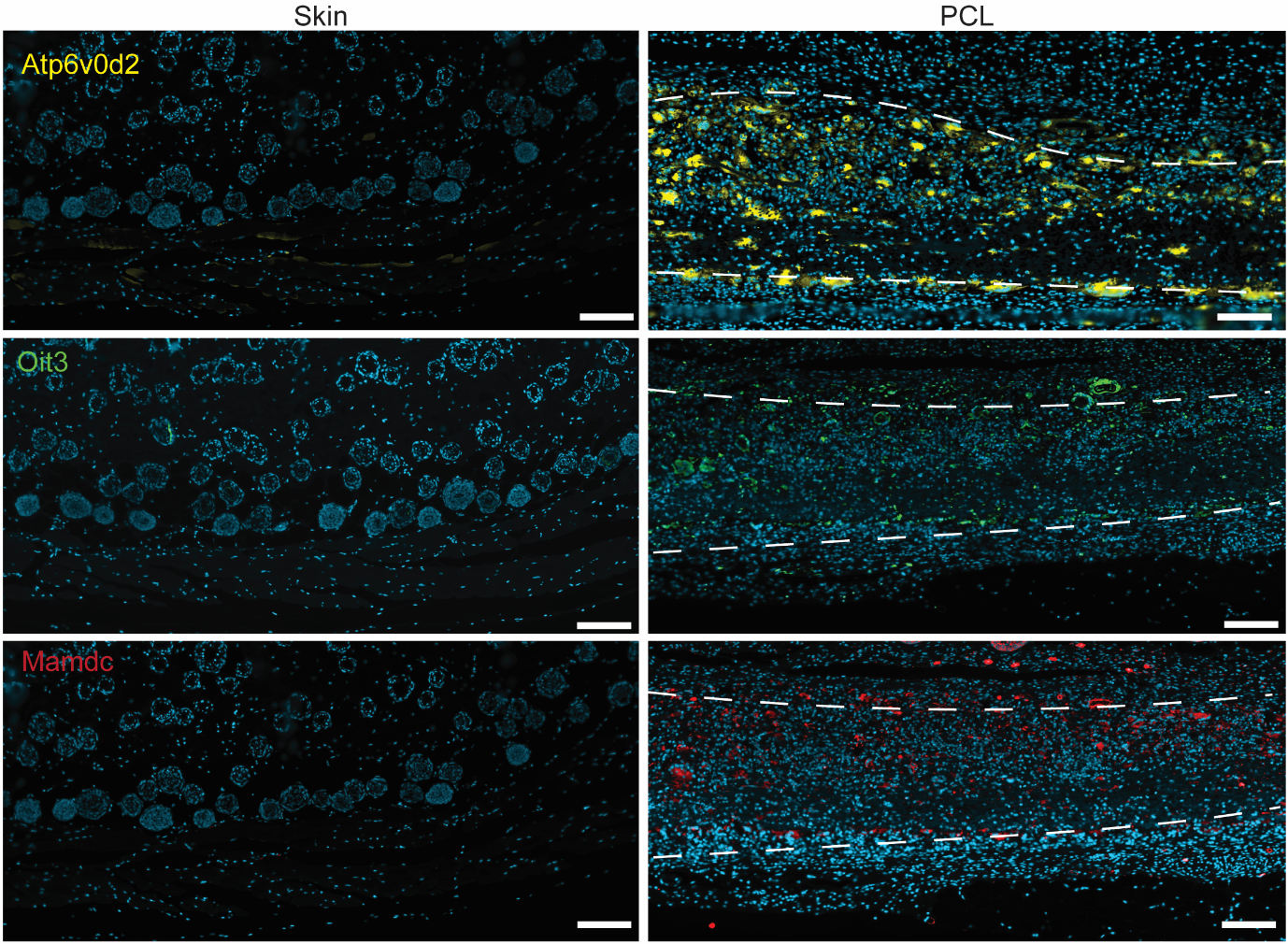
**

**Supp. Figure 4: Representative images of immunohistology of Atp6v0d2, Mamdc2 and Oit3.** (scale bars = 100 µm)

**
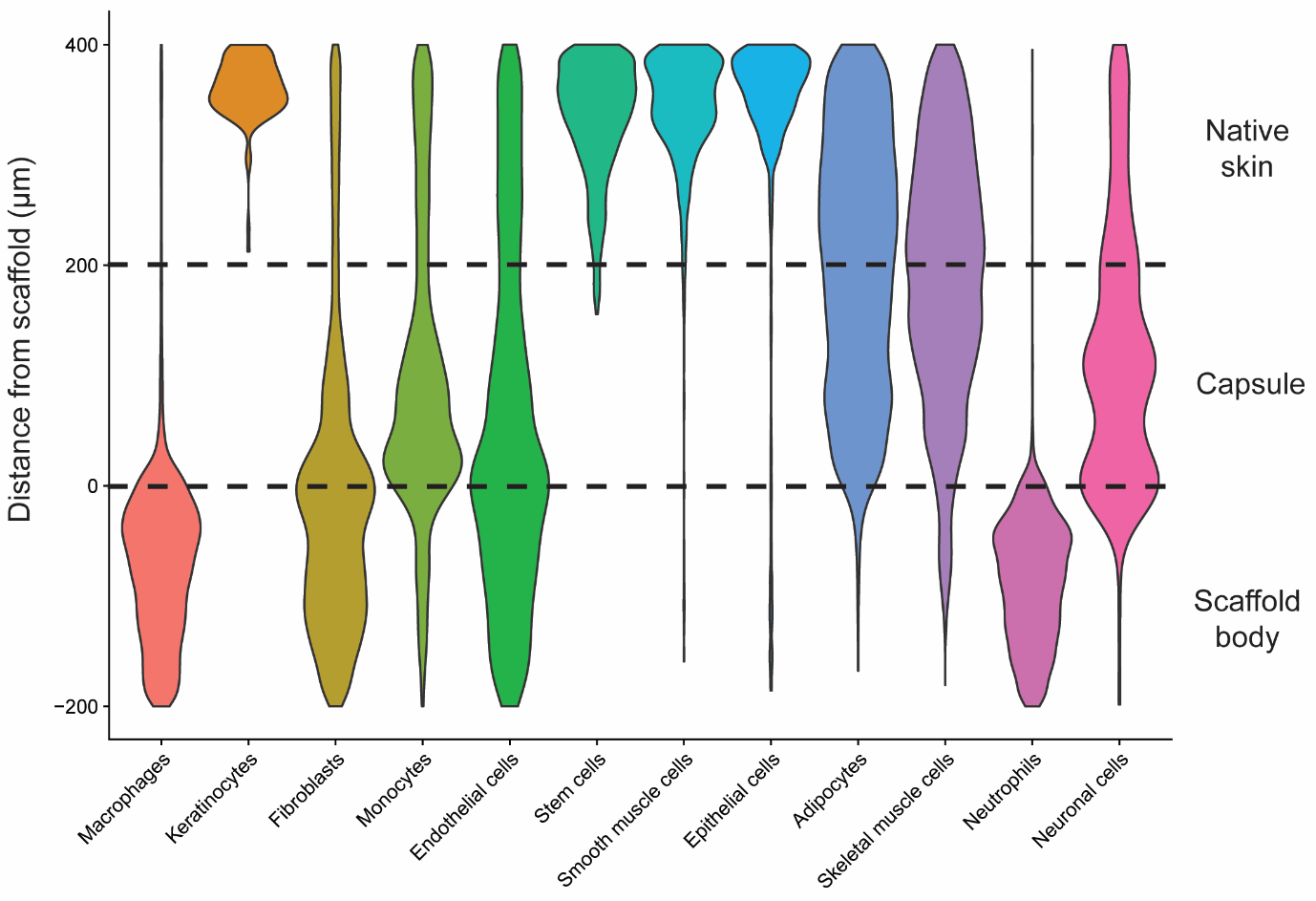
**

**Supp. Figure 5: Perpendicular cell distance from scaffold surface.**

**
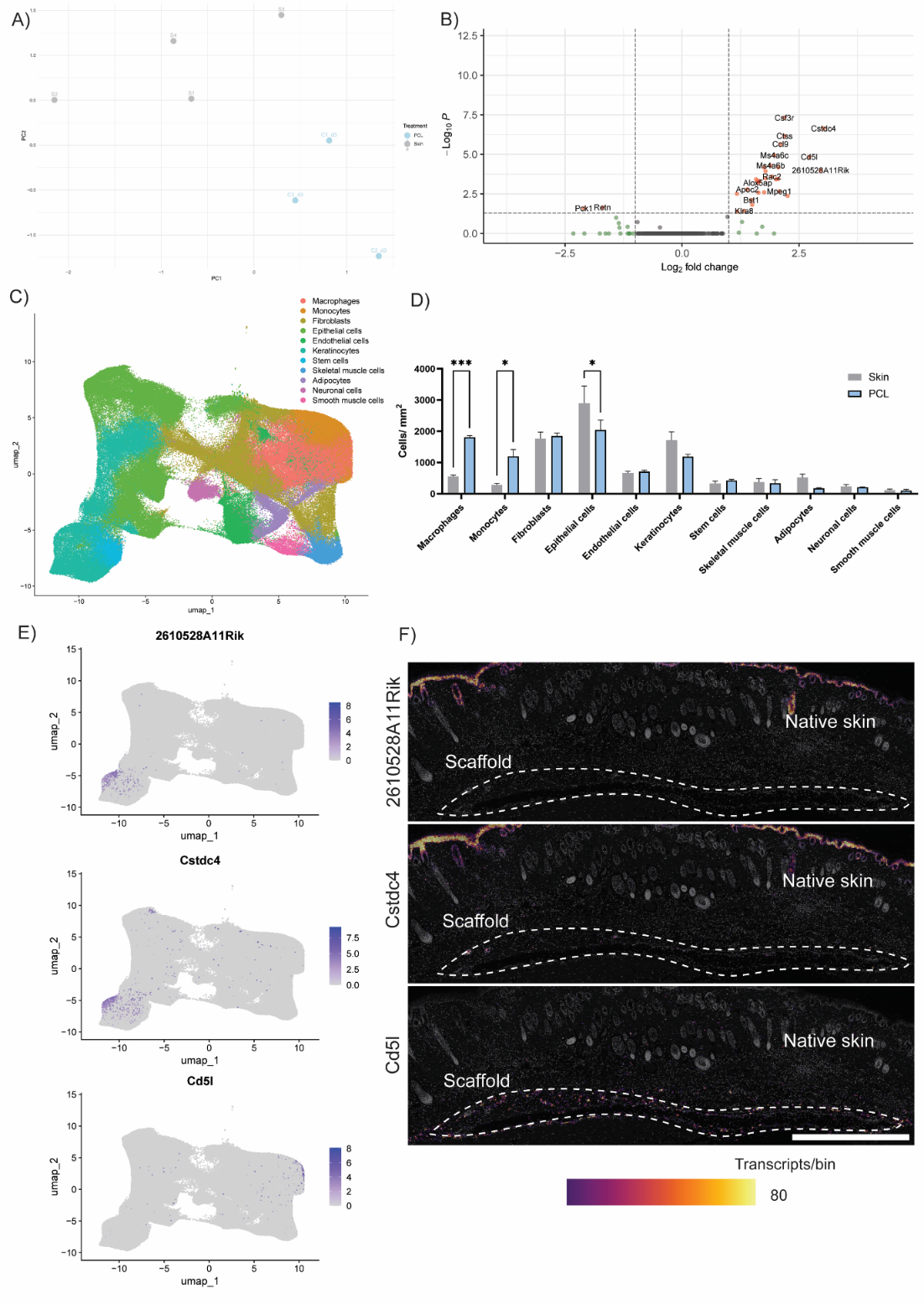
**

**Supp. Figure 6: PCL day 3 post implant.** A) PCA with distinct separation of PCL and control skin samples. C) Volcano plot of differential gene expression between PCL and skin tissue, significantly upregulated genes (log₂FC ≥ 1, –log₁₀p ≥ 2) highlighted in red. D) UMAP visualization of integrated skin and PCL samples using the top 15 principal components. E) Feature plots of 2610528A11Rik, Cstdc4, Cd5l. F) Spatial transcripts of 2610528A11Rik, Cstdc4 and Cd5l mapped onto PCL-implanted tissue. (scale bar = 1000 µm).

**
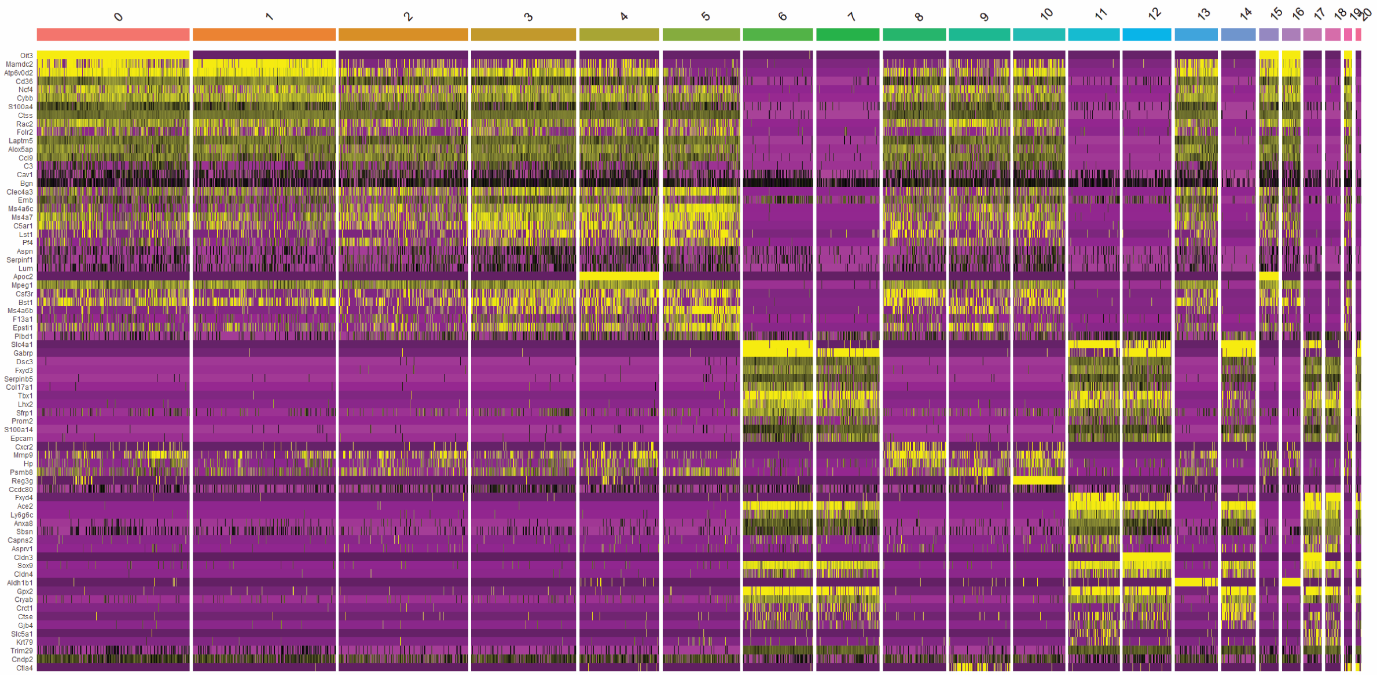
**

**Supp. Figure 7: Heatmap of top 10 upregulated genes for each subcluster of macrophages.**

**
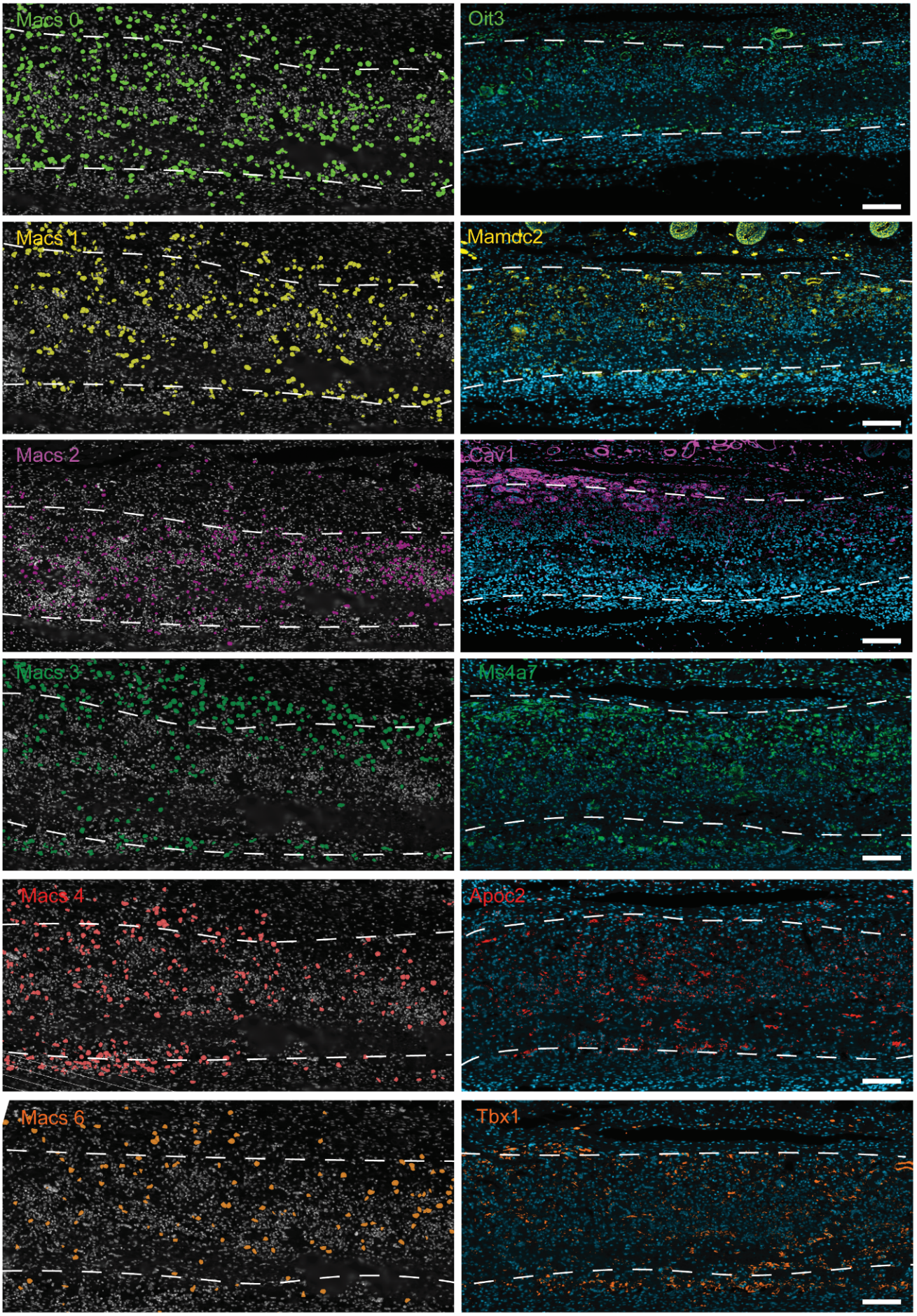
**

**Supp. Figure 8: Validation of PCL-associated macrophage subclusters by immunofluorescence.** Spatial transcriptomic maps (left panels) show the regional localization of each subcluster, while immunofluorescence staining (right panels) shows the corresponding top marker protein; scale bars = 100 µm.

**
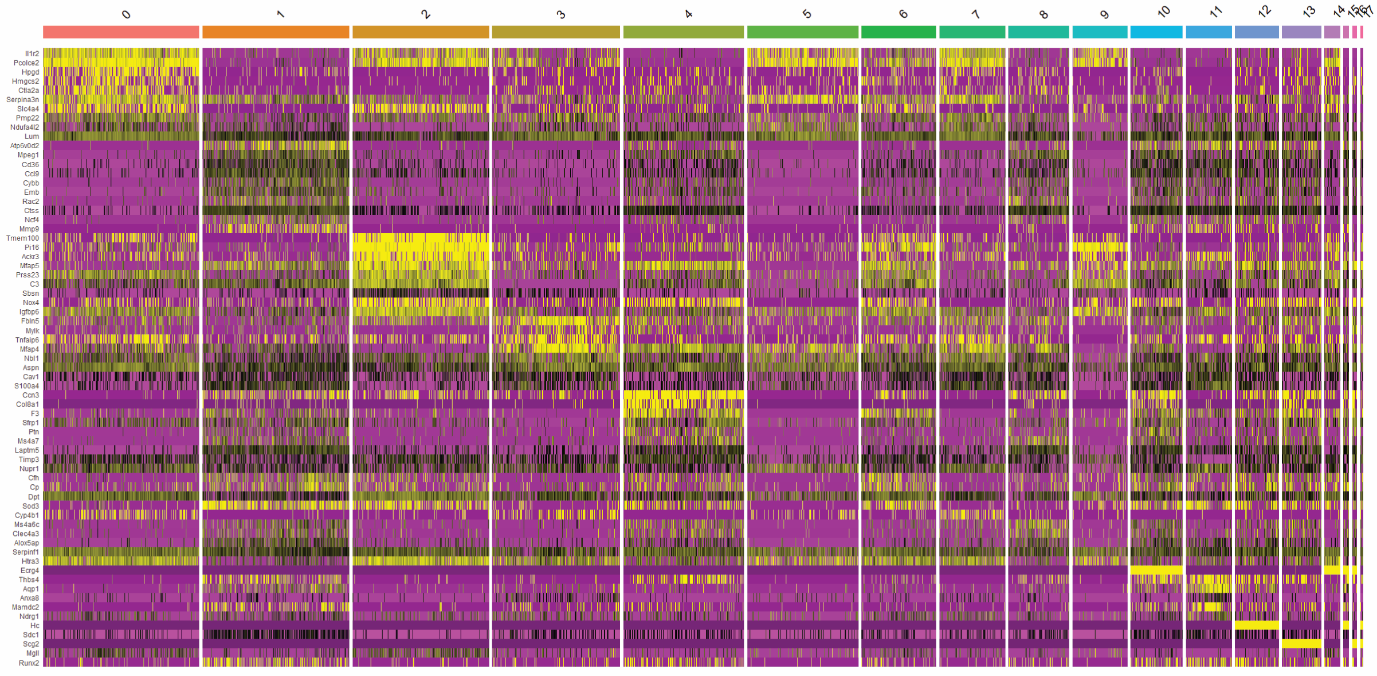
**

**Supp. Figure 9: Heatmap of top 10 upregulated genes for each subcluster of fibroblasts.**

**
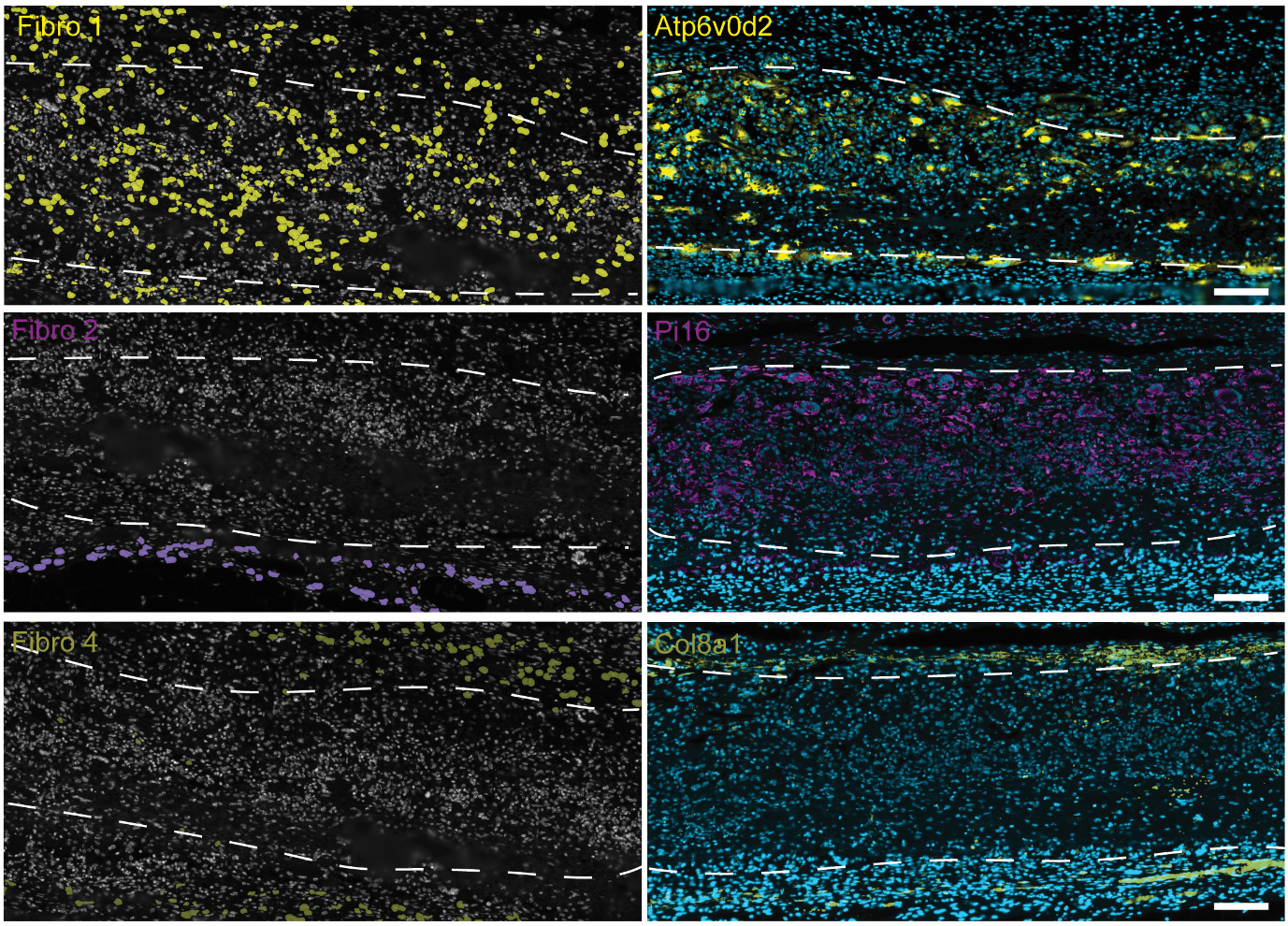
**

**Supp. Figure 10: Validation of PCL-associated fibroblast subclusters by immunofluorescence.** Spatial transcriptomic maps (left panels) show the regional localization of each subcluster, while immunofluorescence staining (right panels) shows the corresponding top marker protein; scale bars = 100 µm.

**
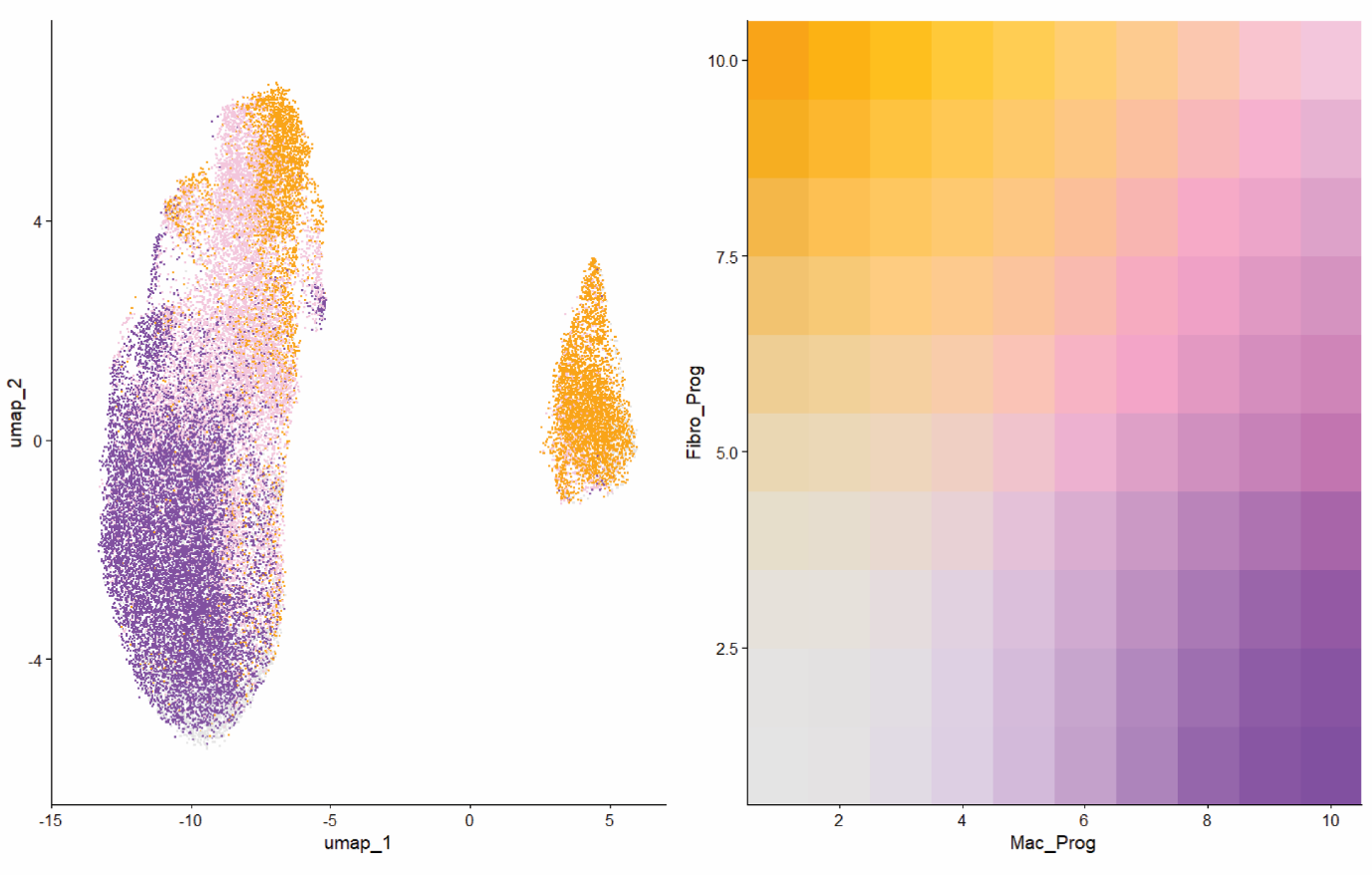
**

**Supp. Figure 11: Featureplot of macrophage-like program and fibroblast-like program.**

**
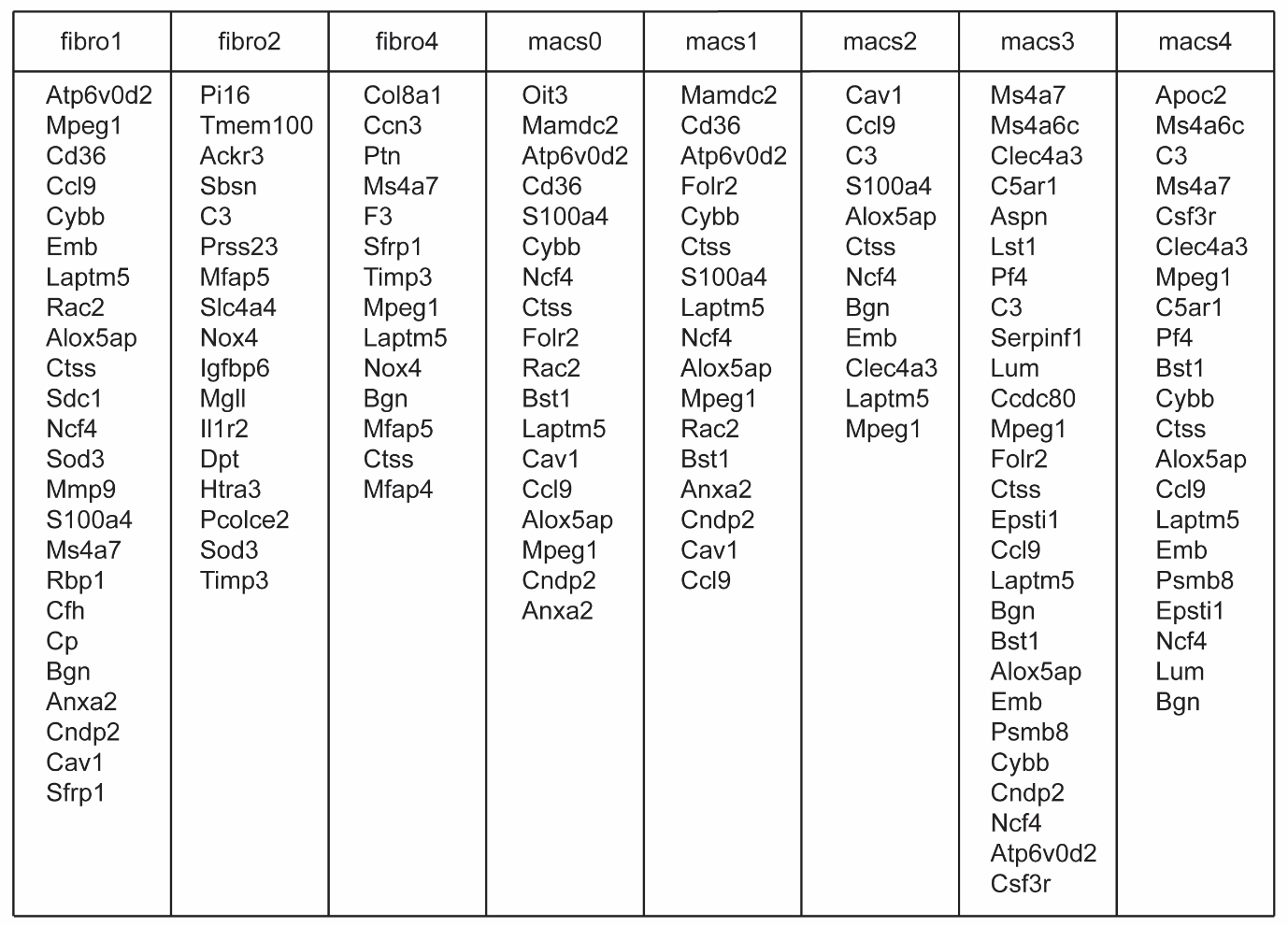
**

**Supp. Table 1: Upregulated genes for subclusters of interest.** Genes were identified with greater than Log_2_FC ≥ 0.25, with expression of at least 50 % of cell population in that cluster.


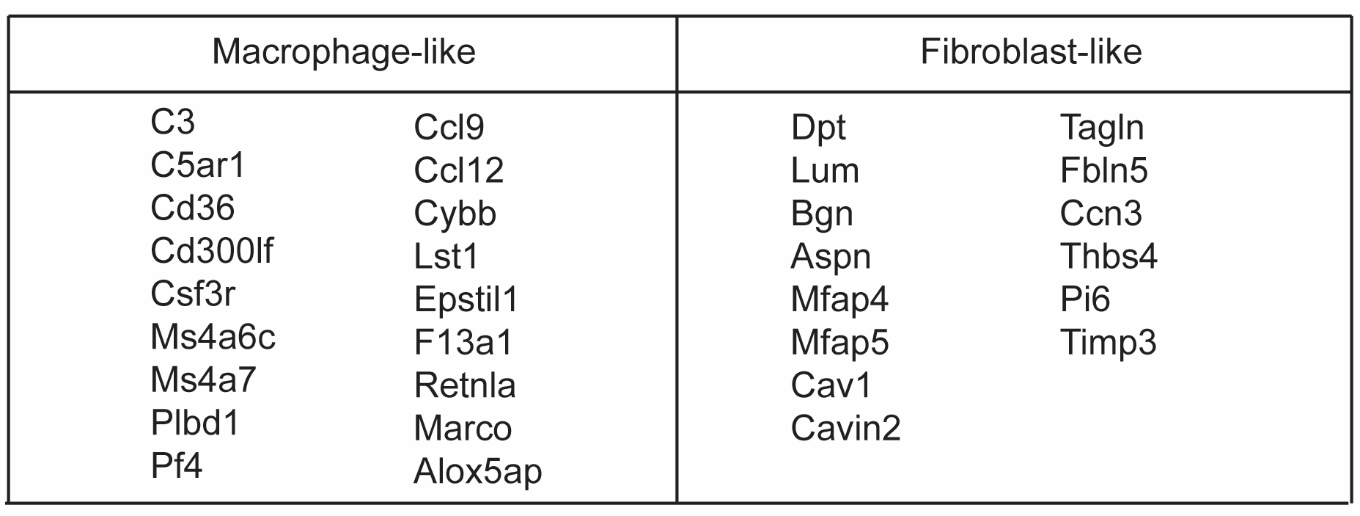


**Supp. Table 2: Genes defining macrophage-like and fibroblast-like program.**
